# Comprehensive analysis across *SMN2* excludes DNA methylation as an epigenetic biomarker for spinal muscular atrophy

**DOI:** 10.1016/j.isci.2025.112461

**Published:** 2025-04-17

**Authors:** Maria M. Zwartkruis, Joris V. Kortooms, Demi Gommers, Martin G. Elferink, Ilaria Signoria, Joyce van der Sel, Paul J. Hop, Ramona A.J. Zwamborn, Robin Geene, Jared W. Green, Hanneke W.M. van Deutekom, Wouter van Rheenen, Jan H. Veldink, Fay-Lynn Asselman, Renske I. Wadman, W. Ludo van der Pol, Gijs W. van Haaften, Ewout J.N. Groen

**Affiliations:** 1 Department of Neurology and Neurosurgery, UMC Utrecht Brain Center, University Medical Center Utrecht, Heidelberglaan 100, 3584 CX Utrecht, the Netherlands; 2 Department of Genetics, University Medical Center Utrecht, Heidelberglaan 100, 3584 CX Utrecht, the Netherlands; 3 Department of Translational Neuroscience, UMC Utrecht Brain Center, University Medical Center Utrecht, Heidelberglaan 100, 3584 CX Utrecht, the Netherlands; 4 Utrecht Sequencing Facility, Center for Molecular Medicine, University Medical Center Utrecht, Heidelberglaan 100, 3584 CX Utrecht, the Netherlands

**Keywords:** Health sciences, Internal medicine, Medical specialty, Medicine, Neurology

## Abstract

Spinal muscular atrophy (SMA) is a severe neurodegenerative disease caused by defects in the *survival motor neuron 1* (*SMN1*) gene. Although disease severity partially correlates with *SMN2* copy number, significant variability in disease severity and treatment response remains unexplained, prompting a search for additional biomarkers. Using native, long-read nanopore and targeted short-read bisulfite sequencing, we analyzed methylation patterns across the 30 kb *SMN2* gene. Our long-read analysis of 29 SMA patients identified tissue-specific variation in *SMN2* intronic regions and the 3′UTR. Further analysis of blood-derived DNA of 365 SMA patients identified no association between *SMN2* methylation and disease severity or treatment response, excluding blood-derived DNA methylation as a predictive biomarker. However, we discovered significant age-associated variation in *SMN2* methylation in intron 1 and the 3′UTR, suggesting a possible role in modifying SMN expression during development and aging. This study provides a framework for detailed methylation analysis in complex genetic regions.

## Highlights

• *SMN2* DNA methylation differs between blood and fibroblasts
• DNA methylation in intron 1 and the 3′UTR of *SMN2* in blood is associated with age
• No link between blood *SMN2* DNA methylation and SMA severity or treatment response

## Graphical abstract

**Figure:**
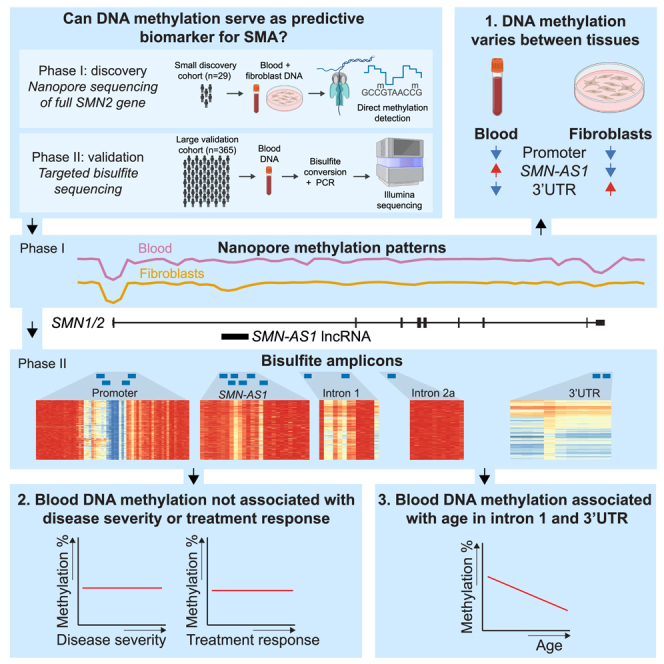

## Introduction

Spinal muscular atrophy (SMA) is a devastating neurodegenerative disease characterized by progressive muscle weakness and atrophy that may cause infantile death or severe childhood disability. The incidence is approximately 1 in 10,000 live births.^1^ SMA is caused by homozygous deletion or mutation of the *survival motor neuron 1* (*SMN1*) gene, which results in insufficient production of the critical and ubiquitously expressed SMN protein.^2^ While SMA patients lack a functional *SMN1* gene, they retain one or more copies of the highly similar *SMN2* gene. The *SMN2* gene can partially compensate for the loss of *SMN1*, but its ability to do so is limited by alternative splicing that excludes exon 7, producing a truncated and unstable form of the SMN protein.^3^^,^^4^^,^^5^ The number of *SMN2* gene copies, ranging from one to six in patients with SMA, inversely correlates with disease severity, with higher copy numbers associated with milder phenotypes.^6^^,^^7^ However, this relationship is not absolute, and significant variability in clinical presentation exists even among patients with the same *SMN2* copy number.^6^^,^^8^ Additionally, the expression of SMN is strongly developmentally regulated through unknown mechanisms, with high pre- and neonatal expression followed by a reduction later in life.^9^^,^^10^ Finally, treatment response to the three currently available gene-targeted therapies varies between patients with equal *SMN2* copy numbers.^11^^,^^12^ These observations suggest that factors beyond *SMN2* copy number may influence SMN protein expression and SMA outcomes.

One potential source of this variability may lie in the epigenetic regulation of *SMN2*, as has often been hypothesized.^7^^,^^11^^,^^13^^,^^14^^,^^15^^,^^16^ DNA methylation is a key epigenetic mechanism that can influence gene expression without altering the underlying DNA sequence.^17^ Previous studies have identified differential methylation patterns in the *SMN2* promoter region between SMA patients with varying disease severities, suggesting that DNA methylation may influence *SMN2* expression.^18^^,^^19^ Furthermore, the methyl-CpG-binding protein MECP2 has been shown to interact with methylated sites in the *SMN2* promoter, potentially regulating *SMN* gene activity.^18^^,^^20^ Several studies investigated genome-wide differential DNA methylation in SMA patients, however, with limited sample sizes.^21^^,^^22^ While these initial findings are intriguing, the existing literature on *SMN2* methylation in SMA is limited, often focusing on a small number of CpG sites within the promoter region and in small patient cohorts. Our overall understanding of variation in DNA methylation in patients with SMA therefore remains limited.

To fully elucidate the role of DNA methylation in the regulation of *SMN2*, a more comprehensive analysis across the entire gene is warranted. In this study, we therefore aimed to provide a detailed characterization of DNA methylation patterns across *SMN2* in a large cohort of patients with SMA, using a combination of long-read Oxford Nanopore Technologies (ONT) sequencing and targeted bisulfite sequencing. We identified lowly methylated CpG sites in the *SMN2* promoter, the transcription site of the long non-coding RNA *SMN-AS1*, several intronic regions, and the 3′UTR of *SMN2*, with tissue-specific differences. No association between DNA methylation and disease severity or treatment response was found, ruling out blood-derived DNA methylation as an epigenetic biomarker of SMA. DNA methylation was significantly associated with age at the *SMN2* 3′UTR and intron 1, suggesting these changes might be involved in the regulation of SMN expression during development and aging.

## Results

### ONT sequencing reveals extensive variation in DNA methylation across *SMN2* and copy-specific differential methylation between *SMN2* haplotypes

**Figure 1:**
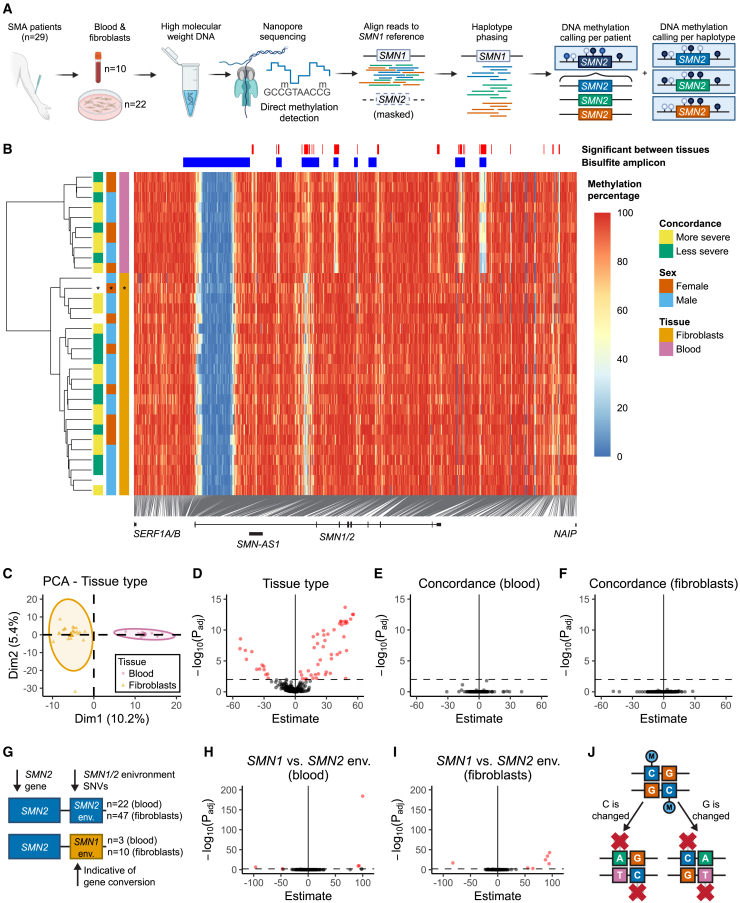
DNA methylation varies extensively throughout the *SMN2* gene and varies between tissues (A) Overview of long-read sequencing and bioinformatics approaches in this study. ONT sequencing with adaptive sampling was performed on high molecular weight DNA of SMA patients. Reads were mapped to *SMN1* and phased into haplotypes using HapSMA.^23^ Methylation percentage per CpG site was determined per patient and per haplotype. Created with Biorender.com. (B) Heatmap of CpG DNA methylation in and around the *SMN2* gene (T2T-CHM13 chr5:71,375,000–71,425,000) in SMA patients with a homozygous *SMN1* deletion and one patient with one copy of *SMN1* with a pathogenic mutation (indicated with an asterisk (∗)). Each row represents one patient, each column represents one CpG site. Hierarchical clustering with the ward.D2 method was performed on the rows. Patient characteristics are shown left of the heatmap. Sites at which methylation significantly differs between tissues (Figure 1D, p_adj_ < 0.01) and the bisulfite amplicons (Figure 2A) are shown above the heatmap. (C) Principal-component analysis (PCA) of the methylation data from (B). Blood samples cluster separately from fibroblast samples. (D) Differential methylation analysis of DNA methylation between fibroblasts (*n* = 22) and blood (*n* = 10). 58 sites are differentially methylated between tissues (p_adj_ < 0.01). (E) Differential methylation analysis of DNA methylation between more severely (*n* = 6) and less severely (*n* = 4) affected patients for blood samples. No differentially methylated sites were found (p_adj_ < 0.01). (F) Differential methylation analysis of DNA methylation between more severely (*n* = 10) and less severely (*n* = 8) affected patients for fibroblast samples. No differentially methylated sites were found (p_adj_ < 0.01). (G) Schematic representation of haplotypes with *SMN1* environment SNVs and *SMN2* environment SNVs. (H and I) Differential methylation analysis of DNA methylation of haplotypes with *SMN1* environment SNVs versus haplotypes with *SMN2* environment SNVs, for blood (H) and fibroblasts (I). Five differentially methylated sites were found in blood and seven sites in fibroblasts. Sample sizes are as indicated in (G). (J) Schematic representation of a methylated CpG site, and likely consequences for methylation if either the C or G changes to another nucleotide: methylation is not present or not detected by the currently used algorithms. See also Figures S1–S3 and Tables S1, S5, S6, and S9.

To comprehensively explore DNA methylation across *SMN2* in SMA patients, we determined CpG methylation status from ONT sequencing data from 10 blood samples and 22 fibroblast samples from 29 patients with varying *SMN2* copy numbers and SMA types (Table S1).^23^ We aligned the sequencing reads to *SMN1* by masking *SMN2* and phased the reads into two to five haplotypes.^23^ We analyzed DNA methylation per patient and per *SMN2* haplotype (Figure 1A) and visualized the methylation percentage per CpG site in a heatmap (Figures 1B and S1). In addition to low methylation of the promoter region, low methylation was also observed in the transcription site of the lncRNA *SMN-AS1*,^14^ the 3′UTR and various other, intronic regions of *SMN2*. Hierarchical clustering (Figure 1B) and principal-component analysis (PCA, Figure 1C) showed that methylation data from fibroblasts and blood clustered separately, as expected based on previous reports showing variation in DNA methylation between tissues.^24^ Fifty-eight sites were differentially methylated (p_adj_ < 0.01) between blood and fibroblasts (Figures 1B and 1D). Because of this, subsequent analyses were performed separately for each tissue. When exploring a possible correlation between DNA methylation and disease severity by SMA type in this small group of samples, no differentially methylated sites were found (Figures 1E and 1F). We explored possible associations between DNA methylation and *SMN2-FL*, *SMN2Δ7*, and *SMN-AS1* RNA expression in fibroblasts. At chr5:71,417,315, DNA methylation was significantly associated with *SMN2Δ7* RNA expression (p_adj_ < 0.01), but the sample size for this analysis was limited (*n* = 8; Figure S2). We and others previously identified multiple single nucleotide variants (SNVs) that can be used to study the genomic environment of individual *SMN* copies and serve as markers of gene conversion^23^^,^^25^ and used these insights to next study DNA methylation per *SMN* haplotype (Figure S3A). When comparing *SMN2* haplotypes with *SMN1* environment SNVs to those with *SMN2* environment SNVs (Figure 1G), we found five differentially methylated CpG sites in blood and seven in fibroblasts (p_adj_ < 0.01), of which four overlapped (Figures 1H, 1I, and S3). However, most of these are at known SNV positions^23^ (Figures S3B and S3C), and these differences are therefore likely caused by a nucleotide variant in the CpG site, resulting in a non-CpG site (Figure 1J). Non-CpG methylation is possible in humans, but its properties and functions are not well known.^26^^,^^27^ Hence, it was not included in the methylation calling algorithms of this study (Methods). Interestingly, although based on a limited number of observations, one of the differentially methylated sites in blood (chr5:71,381,516, promoter region) was not a known SNV site, indicating a possible association between gene environment and methylation status (Figures S3B). In summary, ONT sequencing allowed us to study DNA methylation across the entire *SMN2* gene and led us to identify several regions of interest (ROIs) for further analysis in a larger cohort.

### DNA methylation in the *SMN2* gene can be determined by targeted bisulfite sequencing

**Figure 2:**
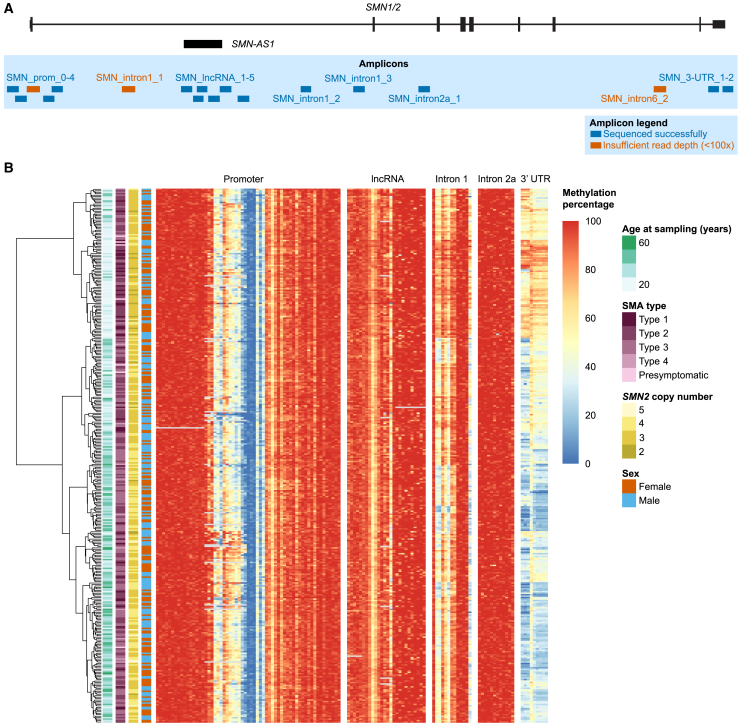
DNA methylation in the *SMN2* gene can be determined by targeted bisulfite sequencing (A) Location of 18 amplicons, 15 of which were sequenced successfully (a total of ∼6.6 kb around *SMN2*). The three amplicons with insufficient read depth for further analyses are also shown. (B) Heatmap of DNA methylation percentage per CpG site determined with targeted bisulfite sequencing. Each row represents one patient, each column represents one CpG site. Hierarchical clustering with the ward.D2 method was performed on the rows. Patient characteristics are shown left of the heatmap. Clustering of patients is mostly based on DNA methylation in intron 1 and the 3′UTR. See also Figures S4–S6 and Tables S2–S4 and S10.

To investigate whether variation in DNA methylation identified by ONT sequencing was linked to disease severity or treatment response, we performed targeted bisulfite sequencing on DNA isolated from blood from 365 SMA patients (Figure S4; Tables 1, S2, and S3). Per patient, we pooled 18 amplicons containing the ROIs identified by ONT sequencing (Figure 2A and Table S4), 15 of which were successfully sequenced by barcoded Illumina sequencing (>100x read depth in at least 90% of patients). We included only CpG sites that were mapped on the intended targets with at least 100x read depth and excluded potential SNV sites (Figure S5A). Median read depth per CpG site was 3,014–37,870x and for every CpG site, 95%–100% of the patients had a methylation call (Figures S6A–S6B). For nine patients, both ONT and bisulfite methylation data from blood was available; methylation percentages showed high correlation between data types (Spearman’s R = 0.77, *p <* 2.2e-16, Figure S6C). Heatmap visualization of methylation percentage per CpG site illustrated that most variation was present in the promoter region and 3′UTR. We performed hierarchical clustering of patients and found that clustering was mostly based on variation in intron 1 and the 3′UTR of *SMN2* (Figure 2B). We performed further dimensionality reduction of the dataset by filtering out CpG sites with little variation in methylation percentage (standard deviation < 5%) and collapsing CpG sites with high correlation (Spearman R > 0.9), resulting in 57 sites for further analysis and statistical testing (Figures S5A and S5B).

**Table 1:** Baseline characteristics of SMA patients sequenced with bisulfite sequencing with homozygous *SMN1* deletion and no c.859G > C variant in *SMN2*

	2x*SMN2*	3x*SMN2*	4x*SMN2*	5x*SMN2*	Overall
Total	15	215	122	7	359

**Sex**					

Male	10 (66.7%)	94 (43.7%)	71 (58.2%)	6 (85.7%)	181 (50.4%)
Female	5 (33.3%)	121 (56.3%)	51 (41.8%)	1 (14.3%)	178 (49.6%)

**SMA type**					

Type 1	15 (100%)	30 (14.0%)	1 (0.8%)	0 (0%)	46 (12.8%)
Type 2	0 (0%)	138 (64.2%)	14 (11.5%)	0 (0%)	152 (42.3%)
Type 3	0 (0%)	43 (20.0%)	96 (78.7%)	5 (71.4%)	144 (40.1%)
Type 4	0 (0%)	0 (0%)	9 (7.4%)	1 (14.3%)	10 (2.8%)
Presymptomatic	0 (0%)	4 (1.9%)	2 (1.6%)	1 (14.3%)	7 (1.9%)

**Age at onset (years)**					

Mean (SD)	0.205 (0.153)	1.08 (0.987)	7.39 (9.45)	11.5 (6.23)	3.30 (6.35)
Median (Min, Max)	0.208 (0, 0.500)	0.917 (0, 9.50)	2.50 (0.458, 43.0)	12.5 (1.00, 19.0)	1.08 (0, 43.0)
Missing	1 (6.7%)	11 (5.1%)	13 (10.7%)	1 (14.3%)	26 (7.2%)

**Age at sampling (years)**					

Mean (SD)	3.57 (4.64)	21.9 (16.9)	35.5 (20.7)	31.2 (14.4)	25.9 (19.6)
Median (Min, Max)	1.86 (0.282, 18.5)	19.6 (0.0438, 64.2)	37.3 (0.189, 79.7)	25.0 (18.0, 52.3)	23.1 (0.0438, 79.7)

### DNA methylation significantly correlates with age at 22 CpG sites across *SMN2*

**Figure 3:**
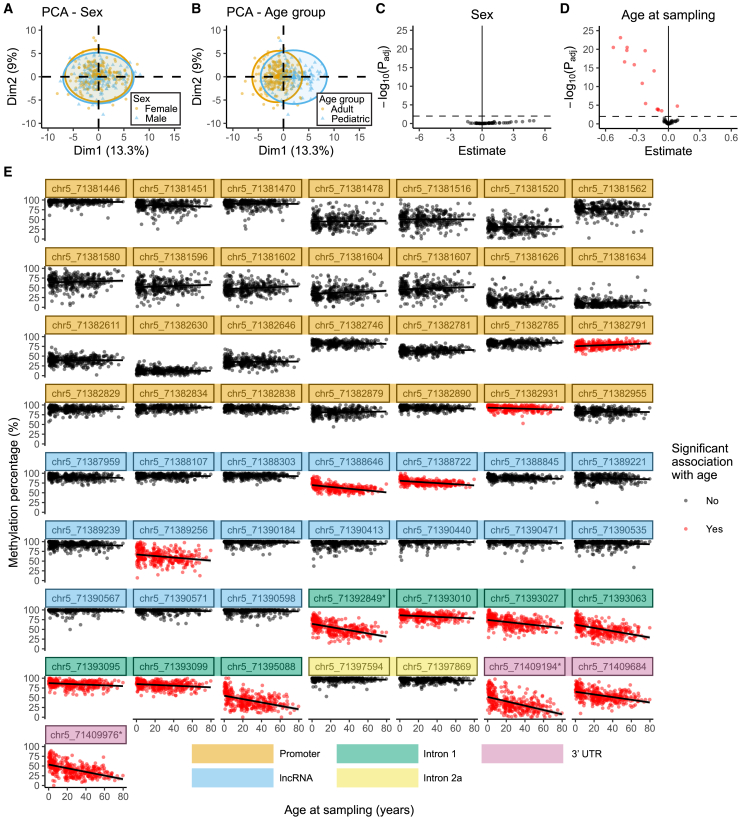
DNA methylation in *SMN2* is significantly associated with age at sampling (A and B) Principal-component analysis (PCA) of the methylation data used for statistical analysis, colored by sex (A) or age (B). Sex clusters overlap completely, whereas age group clusters overlap only partly. (C and D) Differential methylation analysis for sex (C) and age at sampling (D) shown with volcano plots (*n* = 359). Fifteen sites including collapsed sites, corresponding to 22 CpG sites in total, are significantly associated with age (p_adj_ < 0.01). (E) Methylation percentage of all sites analyzed in the differential methylation analysis. Differentially methylated sites from (D) are colored red. Black lines represent linear models between age and methylation percentage, gray shading represents the 95% confidence interval of the linear models. DNA methylation significantly decreased with age in all tested sites in intron 1 and the 3′UTR, and several sites in the promoter and lncRNA and DNA methylation at one site in the promoter significantly increased with age. Sites that were collapsed for statistical analysis (see STAR Methods) are indicated with an asterisk (∗). See also Figures S7–S9 and Table S7.

To explore whether DNA methylation was associated with baseline patient characteristics—such as sex and age—we performed PCA, excluding patients with uncommon genotypes: carriers of an *SMN1* copy with loss-of-function variants or the positive modifier c.859G > C in *SMN2*. Male and female groups completely overlapped (Figure 3A), whereas age group clusters only partly overlapped (Figure 3B). Differential methylation analysis did not yield differentially methylated sites between male and female patients (Figure 3C). In contrast, DNA methylation was significantly associated with age at 15 sites including three collapsed sites (p_adj_ < 0.01, Figure 3D), corresponding to 22 CpG sites in total. Interestingly, DNA methylation percentage increased with age at one site (promoter region) but decreased at all other significantly associated sites in intron 1, the 3′UTR and the lncRNA *SMN-AS1* sequence (Figure 3E). As this association implies that age may be a confounding variable, we corrected for it in our subsequent statistical analyses. The sites associated with age did not contain common nucleotide motifs (Figure S7). Altered DNA methylation may affect binding of CCCTC-binding factor (CTCF), which may in turn affect 3D genome organization and alternative splicing.^28^^,^^29^^,^^30^ However, CTCF binding site motifs^31^ did not overlap with any of the age-related CpG sites in *SMN2*^32^ (Figure S8). To investigate whether age-associated changes in *SMN2* DNA methylation affect *SMN2* mRNA expression, we made use of previously generated expression data from whole blood.^8^ We found that *SMN2-FL* RNA expression was significantly associated with age in patients with three copies of *SMN2*, but not in patients with four copies of *SMN2* (Figure S9A), possibly because this group did not include patients younger than 6 years old and the sharpest decline of SMN expression happens perinatally.^9^ We tested whether there was an association between DNA methylation and *SMN2-FL* expression in patients with three *SMN2* copies. An association can be observed between DNA methylation and *SMN2-FL* expression at most CpG sites associated with age, where methylation appeared to be higher in patients with higher *SMN2-FL* expression (Figure S9B), although these associations were not statistically significant (Figure S9C).

### No association between DNA methylation in *SMN2* and disease severity or treatment response

**Figure 4:**
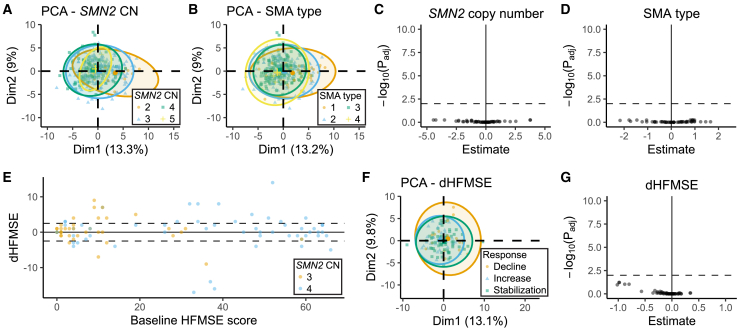
No association was found between *SMN2* DNA methylation and *SMN2* copy number, SMA type or treatment response in SMA patients (A and B) Principal-component analysis (PCA) of DNA methylation on *SMN2* determined by bisulfite sequencing, colored by *SMN2* copy number (A) or SMA type (B). (C and D) Differential methylation analysis for *SMN2* copy number (*n* = 359) (C) and SMA type (*n* = 352) (D). No differentially methylated sites were found (p_adj_ < 0.01). (E) Difference in HFMSE score (dHFMSE) 1.5 years after nusinersen treatment versus before nusinersen treatment in patients with three or four copies of *SMN2*, plotted against baseline HFMSE score. (F) PCA of DNA methylation on *SMN2* determined by bisulfite sequencing, colored by treatment response group based on dHFMSE after nusinersen treatment. Treatment response was divided as follows: decrease: dHFMSE ≤ −3; stabilization: −3 < dHFMSE < 3; increase: dHFMSE ≥ 3. (G) Differential methylation analysis for dHFMSE shown with volcano plots (*n* = 111). No differentially methylated sites were found (p_adj_ < 0.01). See also Figures S10–S12 and Table S8.

To investigate whether DNA methylation was associated with disease severity or treatment response, we first analyzed patients with genetic variants that potentially modify disease severity separately. We found no association between DNA methylation and presence of *SMN1* or the c.859G > C variant in *SMN2* (Figure S10). Patients with these genotypes were excluded from further analyses, since their phenotypes are likely affected by these genetic variants. To test if DNA methylation was associated with *SMN2* copy number, we first performed PCA that suggested there might be differences between copy number groups (Figure 4A). However, this observation was likely confounded by age as mentioned previously, since patients with lower *SMN2* copy number are often sampled at younger ages than patients with higher copy number. Indeed, when correcting for age, none of the tested sites were significantly associated with *SMN2* copy number (Figure 4C). Similarly, no association was found between DNA methylation and copy number of the *NAIP* gene (Figure S11A), which has also been suggested as a potential modifier of SMA, although inconclusively.^13^ No significantly differentially methylated sites were found for disease severity by SMA type (Figures 4B and 4D) or age at onset (Figure S11B). No differentially methylated sites were found when comparing SMA types or ages at onset in patients with three or four *SMN2* copies separately (Figures S11C–S11F). In addition, we tested the differentially methylated CpG sites from previous work^19^ specifically, but did not replicate any of these associations in our cohort (Figure S12). Finally, we compared changes in the commonly used Hammersmith functional motor scale (HFMSE) scores^33^ of a cohort of patients with three or four *SMN2* copies before and 1.5 years after nusinersen treatment initiation (dHFMSE, *n* = 111 Figure 4E). Based on dHFMSE, patients were divided in three treatment response groups: decline, stabilization, and increase. PCA did not show clear clustering of these groups (Figure 4F), and no CpG sites were significantly associated with dHFMSE (Figure 4G). These observations do not completely exclude the possibility that individual or rare changes in DNA methylation may still affect SMA outcomes. However, the absence of associations between variation in *SMN2* DNA methylation and disease severity or treatment response in these analyses excludes *SMN2* DNA methylation as an epigenetic biomarker of SMA in blood.

## Discussion

An important outstanding question in the SMA field is the absence of a clear correlation between phenotype and genotype, even among patients with equal *SMN2* copy number. Variation in DNA methylation of *SMN2* has long been hypothesized as a possible explanation for this discrepancy.^7^^,^^11^^,^^13^^,^^14^^,^^15^^,^^16^ Indeed, several previous studies suggested that variation in DNA methylation could be a potential modifier of SMA,^18^^,^^19^ but these studies were limited by technological possibilities and sample size, and comprehensive analyses of DNA methylation across the complete *SMN2* gene in large SMA patient cohorts had not been done previously. In this study, we addressed this issue by combining long-read ONT sequencing to first discover variability in DNA methylation across the complete *SMN2* gene in a limited group of patients, followed by targeted bisulfite sequencing across the identified sites in a large cohort of 365 SMA patients. We identified variable DNA methylation at multiple sites, including the *SMN2* 3′UTR and the intronic transcription site of lncRNA *SMN-AS1*. DNA methylation was significantly associated with age at all tested sites in intron 1 and the 3′UTR of *SMN2*, two CpG sites in the promoter and three CpG sites in the transcription site of lncRNA *SMN-AS1*. However, DNA methylation was not significantly associated with *SMN2* copy number, disease severity, treatment response, or *SMN2-FL* mRNA expression. In summary, our results exclude variation in blood-derived DNA methylation in *SMN2* as an epigenetic biomarker of clinical SMA outcomes but highlight DNA methylation as a possible modifier of SMN expression.

We did not identify methylation differences in the *SMN2* promoter between SMA types in patients with three copies of *SMN2* as reported previously.^19^ This previously reported difference may have been observed due to a lower sample size (*n* = 35 vs. *n* = 211 in our study) or lenient multiple testing correction due to inclusion of a much smaller number of CpG sites. Another previous study specifically investigated DNA methylation in a group of patients with two *SMN2* copies and either SMA type 1 or SMA type 3.^18^ We were unable to replicate these results as the number of patients with two *SMN2* copies in our cohort was limited. In addition, the combination of two *SMN2* copies and SMA type 3 is extremely uncommon, and it cannot be ruled out that the patients included in the previous study were carriers of rare positive modifiers, such as c.859G > C^34^ or c.835-44A > G,^35^ which were not commonly characterized at the time that study was performed. We did not find associations between DNA methylation and disease severity in blood as accessible biomarker tissue, but DNA methylation may still play a role in disease-relevant inaccessible tissues, such as the spinal cord. Although we did not find DNA methylation in blood measured before treatment initiation to be associated with treatment response in this study, there is still a possibility that epigenetic marks might be involved in the regulation of exon 7 inclusion during treatment.^36^ Therefore, longitudinal epigenetic studies may still hold promise for identifying additional targets to strengthen the ability of splice modifiers to increase SMN expression.

In contrast to limited variability in DNA methylation in the *SMN2* promoter region, we observed significant variation in the *SMN2* 3′UTR. Previous studies have associated 3′UTR methylation of other genes with both increased gene expression,^37^^,^^38^^,^^39^ decreased gene expression,^40^^,^^41^^,^^42^^,^^43^^,^^44^^,^^45^ or both.^46^^,^^47^ Therefore, it is likely that 3′UTR DNA methylation can affect gene expression in different ways for different genes, and through different mechanisms. Firstly, the binding of methyl-CpG binding domain (MBD) proteins may be altered by differential methylation.^48^ Secondly, the recognition of polyadenylation signals may be influenced by DNA methylation in the 3′UTR. Specifically, lowered DNA methylation enables binding of CCCTC-binding factor (CTCF) and recruitment of the cohesin complex. This interaction leads to the formation of chromatin loops, which subsequently favor the use of proximal polyadenylation sites.^49^ Alternative cleavage and polyadenylation (APA) can regulate protein expression through several mechanisms, including mRNA stability, mRNA localization, and mRNA translation.^50^ Thirdly, DNA methylation influences alternative splicing. DNA demethylation of the 3′UTR of the *TARDBP* gene reduced alternative splicing and increased *TARDBP* mRNA expression.^44^ The effect of decreased DNA methylation on alternative splicing may be mediated by binding of CTCF, which hinders elongation by RNA polymerase II, which may result in the inclusion or exclusion of specific exons in the mature mRNA.^28^^,^^29^ However, CTCF binding site motifs do not overlap with any of the age-related CpG sites in *SMN2*, limiting the likelihood that CTCF binding is affected by DNA methylation during aging (Figure S8). Possibly through similar mechanisms, DNA methylation in intron 1 showed an inverse correlation with gene expression across tissues and species^51^ and for *MECP2* specifically, methylation of the promoter and first intron impacted *MECP2-E1* and *E2* isoform levels.^52^ In the *SMN* genes specifically, Marasco et al. showed that a nusinersen-like ASO promotes chromatin-silencing mark H3K9me2, slowing down RNA polymerase II elongation and inhibiting exon 7 inclusion.^36^ Reduced DNA methylation in gene bodies is linked to increased chromatin accessibility,^53^ and increased DNA methylation and H3K9 methylation with heterochromatin.^54^ Therefore, reduced DNA methylation at the 3′ end *SMN2* could potentially be associated with faster RNA polymerase II elongation and increased exon 7 inclusion. Although we identified no significant association between *SMN2-FL* expression and DNA methylation in blood, further association analyses between DNA methylation, histone modifications and RNA sequencing in other tissues, for example with long-read sequencing,^55^ may indicate whether epigenetic marks affect splicing and expression of specific exons and isoforms of the *SMN* gene.

DNA methylation is associated with age across mammalian tissues, and this association can be either positive or negative, varying per CpG site.^56^ Our current study identified several CpG sites, mostly located in intron 1 and the 3′UTR of *SMN2*, which were associated with age in our patient cohort. For most sites, we observed that higher age was associated with reduced levels of DNA methylation. A similar pattern was found in the *TARDBP* gene in human motor cortex of healthy subjects and ALS patients, and accelerated DNA demethylation in ALS was associated with earlier disease onset.^44^ Therefore, it would be interesting to determine whether age-related decrease of DNA methylation in *SMN2* is also present in healthy subjects. Analyzing this in large control cohorts, however, is challenging as short-read sequencing of bisulfite converted DNA samples does not allow to distinguish *SMN1* and *SMN2* in persons who carry both genes in contrast to SMA patients who only carry copies of *SMN2*. This may become possible in the future when long-read sequencing allowing haplotype-based analysis becomes more routine and scalable. Previous research has shown that SMN protein levels decline rapidly during the perinatal period in *postmortem* spinal cord.^9^ Similarly, *SMN* mRNA and SMN protein expression have been reported to decrease with age in peripheral blood mononuclear cells^10^ but remain relatively stable in primary fibroblasts derived from adult patients.^10^^,^^57^ These findings highlight the potential differences in SMN requirements between young children and adult patients, with potential implications for treatment requirements. While infants and young children may benefit from therapies that maximize SMN expression especially during the first few months of life, this may change in adulthood when patients may have different needs in terms of maintaining sufficient SMN levels as they age. The age-related changes in *SMN2* methylation patterns observed in our study could represent an important consideration for the development of epigenetic therapies that aim to modulate SMN expression across the lifespan.

Current genome-wide epigenetic therapies approved for clinical use include DNA methyltransferase (DNMT) inhibitors, histone deacetylase (HDAC) inhibitors, isocitrate dehydrogenase inhibitors, and enhancer of zeste homolog 2 inhibitors.^58^ There is a complex interplay between DNA methylation and other histone modifications^59^^,^^60^. Histone acetylation close to the *SMN* transcriptional start site has been shown to decrease during mouse development, concomitantly with a decrease in SMN transcript and protein levels,^61^ leading to HDAC inhibition as a possible therapeutic strategy for SMA^62^; however, clinical trials showed limited or no effect, possibly due to diverse responsiveness.^63^^,^^64^^,^^65^ However, more recent studies in mice and SMA patient fibroblasts show promising additive effects of HDAC inhibitors on nusinersen-like ASOs,^36^^,^^66^^,^^67^ likely through removal of the heterochromatin “roadblock” promoted by these ASOs.^36^ Moreover, targeted epigenetic editing technologies are emerging^68^: e.g., catalytically inactive Cas9 (dCas9) that can be targeted to virtually any genomic locus using a gRNA complementary to the target sequence, fused to the catalytic domain of TET1 (TET1CD) for demethylation^69^ or the catalytic domain of DNA methyltransferase 3A (DNMT3A) to increase methylation.^70^ For SMA, demethylation of the 3′UTR with dCas9-TET1CD may increase *SMN2* exon 7 inclusion. However, several challenges still exist for clinical translation of these novel technologies, such as delivery methods, durability, and specificity.^68^ Since our results show an inverse correlation between age and DNA methylation in intron 1 and the 3′UTR, this potentially means that younger patients have a more closed chromatin state, and therefore lower exon 7 inclusion, as discussed in the previous paragraph.^36^ Speculatively, this could mean that promoting a euchromatin state with HDAC inhibitors or targeted methods, in addition to existing splice modifiers, may have a larger additive effect in younger age groups but this would require significant further research efforts.

In conclusion, we used ONT long-read sequencing to reveal extensive variation in DNA methylation across the *SMN2* gene, with low methylation levels observed not only in the promoter region but also in the transcription site of *SMN-AS1*, the 3′UTR, and various intronic regions. With targeted bisulfite sequencing of 365 SMA patients, no associations were found between blood-derived DNA methylation and sex, *SMN2* copy number, disease severity or treatment response, excluding DNA methylation in *SMN2* as an epigenetic biomarker of clinical SMA outcomes. We identified 22 CpG sites across *SMN2* where blood-derived DNA methylation was significantly associated with age, highlighting DNA methylation as a possible modifier of SMN expression during development and aging.

### Limitations of the study

Our study aimed to investigate DNA methylation in the *SMN2* gene as potential biomarker for SMA disease severity and treatment response. One limitation is that our study mostly includes patients with three or four *SMN2* copies, and that the number of patients with two or five *SMN2* copies is limited. Therefore, our conclusions are mostly applicable to the three- and four-copy groups. Secondly, environmental exposures, such as air pollution, smoking, and alcohol intake can affect DNA methylation, and may therefore form confounding factors in methylation studies,^71^^,^^72^ however, data to correct for these variables in our study was unavailable. Thirdly, we used blood-derived DNA as a proxy for methylation patterns in the spinal cord. As DNA methylation differs between tissues, these tissues may not be representative for the spinal cord. However, accessing spinal cord tissue in SMA patients is only possible *postmortem* and therefore of limited use for prognostic biomarker studies. Although technically challenging, cell-free DNA (cfDNA) in cerebrospinal fluid (CSF) may contain DNA derived from degenerating motor neurons and may therefore be used for studying DNA methylation. Longitudinal analysis of DNA methylation during treatment may provide a more dynamic understanding of its potential role in SMA progression and treatment response.

## Resource availability

### Lead contact

Requests for further information and resources should be directed to and will be fulfilled by the lead contact, Ewout J.N. Groen (UMC Utrecht, Heidelberglaan 100, 3584 CX Utrecht, the Netherlands; e.j.n.groen-3@umcutrecht.nl, +31 8875 73834).

### Materials availability

This study did not generate any new unique reagents.

### Data and code availability

• Data: Long-read nanopore and bisulfite sequencing data generated for SMA patients included in this study, including the clinical metadata, cannot be made publicly available due to legal restrictions and patient confidentiality. However, academic request to reuse the data will be granted if the research question aligns with the original informed consent and IRB approval. Summary statistics for ONT and bisulfite methylation data are available with the publication (Tables S9 and S10).
• Code: The main codes utilized for the analysis are available on GitHub: https://github.com/ghaaften/SMA_DNA_methylation.^71^
• Other items: Any additional information required to reanalyze the data reported in this work is available from the lead contact upon request.

## Acknowledgments

We thank the patients and their families for their participation in this study. This work was supported by grants from Stichting Spieren voor Spieren (to W.L.v.d.P.), the European Union’s Horizon 2020 Research and Innovation Program under the Marie Skłodowska-Curie grant (H2020 Marie Skłodowska-Curie actions) agreement no. 956185 (SMABEYOND ITN to W.L.v.d.P. and E.J.N.G.) and Prinses Beatrix Spierfonds (W.OB21-01 to E.J.N.G.). This project has received funding from the 10.13039/501100000781European Research Council (ERC) under the European Union’s Horizon 2020 research and innovation program (grant agreement no 772376 - EScORIAL). We acknowledge the Utrecht Sequencing Facility (USEQ) for providing sequencing service and data. USEQ is subsidized by the University Medical Center Utrecht and The Netherlands X-omics Initiative (NWO project 184.034.019). We thank Charlotte Sumner and Stephen Brown (John Hopkins University) for helpful suggestions for optimizing *SMN-AS1* quantification. We thank Lotte Geerlofs for her efforts in optimizing *SMN-AS1* quantification.

## Author contributions

Conceptualization, M.M.Z., M.G.E., D.G., E.J.N.G., and G.W.v.H.; experimental studies, M.M.Z., I.S., J.V.K., J.W.G., and R.G.; bioinformatics and data analysis, M.M.Z., M.G.E., D.G., H.W.M.v.D., J.v.d.S., R.A.J.Z., P.J.H., and W.v.R.; clinical data and patient material, F.-L.A., R.I.W., and W.L.P.; writing–original draft, M.M.Z., E.J.N.G., and G.W.v.H.; writing–review, all; resources, E.J.N.G. and W.L.v.d.P.; supervision, E.J.N.G., J.H.V., W.L.v.d.P., and G.W.v.H.; funding acquisition, E.J.N.G., G.W.v.H., and W.L.v.d.P.

## Declaration of interests

W.v.R. has sponsored research agreements with Biogen and Astra Zeneca. J.H.V. reports to have sponsored research agreements with Biogen, Eli Lilly and Astra Zeneca.

## STAR★Methods

### Key resources table

**Table:** 

REAGENT or RESOURCE	SOURCE	IDENTIFIER
**Critical commercial assays**		

SALSA MLPA Probemix P021 SMA Version B1	MRC Holland	P021-B1
Bl chemagic DNA Blood 4k kit	Revvity	CMG-1074
Quant-iT™ 1X dsDNA BR Assay	Invitrogen	Q33267
Fragment Analyzer Genomic DNA 50kb Kit	Agilent	DNF-467
EZ-96 DNA Methylation-Lightning Kit	Zymo	D5033
KAPA HiFi HotStart Uracil+ ReadyMix (2X)	Roche	7959052001
Sybr Safe	Fisher Scientific	#10328162
High Sensitivity D1000 ScreenTape	Agilent	5067-5584
High Sensitivity D1000 Reagents	Agilent	5067-5585
NEBNext Q5 Hot Start HiFi PCR Master Mix	NEB	M0544L
Qubit dsDNA High Sensitivity kit	Thermo Fisher Scientific	#Q32854
RNeasy mini kit	Qiagen	74104
DNase I	Thermo Scientific	EN0521
High-capacity cDNA reverse transcription kit	Applied Biosystems	4368814
2x ddPCR Supermix for probes (no dUTP)	BioRad	186-3024

**Oligonucleotides**		

Primers for bisulfite sequencing	This paper	Table S4
Illumina DNA/RNA UD Indexes Sets A-D	Illumina	20091654, 20091656, 20091658, 20091660
ddPCR primers and probes	Wadman et al.^10^ Ramos et al.^9^ d’Ydewalle et al.^14^ Signoria et al.^57^ This paper	Table S5

**Software and algorithms**		

Zymo Bisulfite Primer Seeker	Zymo Research^73^	https://zymoresearch.eu/pages/bisulfite-primer-seeker
Fiji software (version 1.54)	Schindelin et al.^74^	https://imagej.net/software/fiji/
QuantaSoft Software	BioRad	1864011
Guppy v6.1.2	ONT^75^	https://nanoporetech.com/software/other/guppy
HapSMA v1.0.0	Zwartkruis et al.^23^^,^^76^	https://github.com/UMCUGenetics/HapSMA
modbam2bed v1.0	ONT^77^	https://github.com/epi2me-labs/modbam2bed
methylseq v2.6.0	Ewels et al.^78^	https://nf-co.re/methylseq/2.6.0/
R4.4.0	R Core Team^79^	https://www.r-project.org/
pheatmap v1.0.12 package	Kolde^80^	https://cran.r-project.org/web/packages/pheatmap/
FactoMineR v2.11 package	Lê, Josse, and Husson^81^	https://cran.r-project.org/web/packages/FactoMineR/
factoextra v1.0.7 package	Kassambara and Mundt^82^	https://cran.r-project.org/web/packages/factoextra/
pwr v1.3-0 package	Champely et al.^83^	https://cran.r-project.org/web/packages/pwr/
bedtools v2.30.0	Quinlan and Hall^84^	https://github.com/arq5x/bedtools2
FIMO	Grant, Bailey, and Noble^32^	https://meme-suite.org/meme/doc/fimo.html
WebLogo	Crooks et al.^85^	https://weblogo.threeplusone.com/create.cgi
SMA DNA Methylation (GitHub: codes used for analyses in this manuscript)	Zwartkruis and Gommers^86^	https://github.com/ghaaften/SMA_DNA_methylation

### Experimental model and study participant details

#### Study population

We included 370 SMA patients from our single-center prevalence cohort study in the Netherlands, as detailed in Tables 1 and S1–S3. Twenty-nine of these patients were included for Oxford Nanopore Technologies (ONT) sequencing and 365 patients were included for bisulfite sequencing; 24 patients overlap between ONT and bisulfite sequencing datasets. Although ancestry data collection is not included in our study protocol, most patients are of Dutch ancestry. The study protocol (09307/NL29692.041.09) was approved by the Medical Ethical Committee of the University Medical Center Utrecht and was registered in the Dutch registry for clinical studies and trials.^87^ Written informed consent was obtained from all adult participants, as well as from parents or guardians for patients under 18 years of age. *SMN1*, *SMN2*, and *NAIP* copy numbers were quantified using multiplex ligation-dependent probe amplification (MLPA) (MRC Holland, SALSA MLPA Probemix P021 SMA Version B1) following the manufacturer’s instructions. The clinical classification of SMA type was conducted based on motor milestones and age at onset, with type 1 as non-sitters, type 2 as sitters, and type 3 and 4 as walkers.^88^ To capture the broad genotypic and phenotypic diversity within the SMA population, the study included patients with *SMN2* copy numbers ranging from two to five, and SMA types ranging from 1b to 4. Whole blood samples were collected in EDTA tubes for DNA extraction, and 3 mm dermal biopsies were obtained for the generation of primary fibroblasts. For 111 patients with three or four *SMN2* copies and receiving nusinersen treatment, Hammersmith Functional Motor Scale – Expanded (HFMSE)^33^ scores were available that had been determined before treatment start and 1.5 years after treatment initiation. For PCA, we stratified patients based on treatment response: decrease for dHFMSE≤-3; stabilization for -3<dHFMSE<3; increase for dHFMSE≥3.^89^

### Method details

#### DNA extraction and bisulfite conversion

For ONT sequencing of SMA patients, high molecular weight (HMW) DNA was extracted from fresh or frozen whole EDTA blood (n=7), cultured primary dermal fibroblasts (n=19) or both tissues (n=3) using the Monarch® HMW DNA Extraction Kit for Cells & Blood (New England Biolabs (NEB), T3050L) with lysis agitation at 1400 rpm. DNA concentration was measured using the Qubit™ dsDNA Quantification Broad Range Assay (Invitrogen, Q32853) and purity was determined on a spectrophotometer (Nanodrop 2000, Thermo Scientific). For bisulfite sequencing, DNA was extracted from whole EDTA blood with the Bl chemagic DNA Blood 4k kit (Revvity, CMG-1074). DNA concentration was quantified with the Quant-iT™ 1X dsDNA BR Assay (Invitrogen, Q33267). Genomic Quality Number (GQN) was determined with the Fragment Analyzer Genomic DNA 50kb Kit (Agilent, DNF-467). 500ng of DNA was bisulfite-converted using the EZ-96 DNA Methylation-Lightning Kit (Zymo Research, D5033) according to the manufacturer’s instructions. Concentration of converted DNA was measured with a spectrophotometer (Thermo Scientific, Nanodrop 2000) with the ssDNA setting.

#### Polymerase chain reaction for bisulfite amplicon sequencing

Primers to amplify the top strand of bisulfite-converted DNA were designed with the Zymo Bisulfite Primer Seeker (https://zymoresearch.eu/pages/bisulfite-primer-seeker) ^73^ with default options and intended product sizes of 450-550bp. Primers included a sequencing adapter-compatible overhang (5′-TCGTCGGCAGCGTCAGATGTGTATAAGAGACAG-3′ to 5′ end of the forward primer, 5′-GTCTCGTGGGCTCGGAGATGTGTATAAGAGACAG-3′ to 5′ end of the reverse primer). Primer sequences (Integrated DNA Technologies) and amplicon-specific conditions are listed in Table S4. Eighteen PCRs were performed on each bisulfite-converted patient DNA sample. Each 10uL PCR reaction contained 2.4μL PCR-grade water, 5μL KAPA HiFi HotStart Uracil+ ReadyMix (2X) (Roche, 7959052001), 0.3μL of each primer (10μM), and a variable amount of DNA optimized for each reaction (Table S4). The PCR was run using a Biorad T100 thermocycler (#1861096) with the following protocol: 95°C for 3 minutes; 36 cycles of 98°C for 30 seconds, annealing at variable temperatures for 15 seconds (ramp rate 0.5°C/s), followed by 72°C for 15 seconds (ramp rate 1.1°C/s); and a final elongation at 72°C for 1 minute. All 18 amplicons were visualized on a 2% agarose gel containing Sybr Safe (Fisher Scientific, #10328162) for a subset of eight randomly selected samples and imaged on a Biorad ChemiDoc™ MP Imaging System (#12003154). Band intensity was quantified with Fiji software (version 1.54).^74^ Relative band intensity was used for determining the pooling ratio for amplicon pooling, e.g. a relative brightness of 2x resulted in a pooling ratio of 0.5x. All 18 amplicons were pooled per patient and subsequently cleaned with AMPure XP beads at a ratio of 0.65x to eliminate PCR reagents and small DNA fragments. A subset of samples was run on the Agilent 2200 TapeStation (#G2965A) with Agilent High Sensitivity D1000 ScreenTape (5067-5584) and High Sensitivity D1000 Reagents (5067-5585) to check for overamplification.

#### Library preparation and Illumina sequencing

A second PCR was performed on each patient sample containing 18 pooled amplicons to attach Illumina DNA/RNA UD Indexes Sets A-D (Illumina, 20091654, 20091656, 20091658, 20091660) barcodes to the compatible overhangs that were included in the first PCR (see above). Each 25uL PCR reaction contained 5.5μL PCR-grade water, 12.5μL NEBNext Q5 Hot Start HiFi PCR Master Mix (NEB, M0544L), 2.5μL of unique Illumina® DNA/RNA UD Index, and 2.5ng of DNA in a volume of 2μL. The PCR was run in a Biorad T100 thermocycler with the following steps: 98°C for 30 seconds; six cycles of 98°C for 10 seconds followed by 65°C for 75 seconds; and a final elongation at 65°C for five minutes. The PCR products were purified with AMPure XP beads at a ratio of 0.8x. A subset of samples was measured on the Agilent 2200 TapeStation with Agilent High Sensitivity D1000 ScreenTape and High Sensitivity D1000 Reagents to check if the Illumina DNA/RNA UD Indexes had successfully annealed. The concentration of a subset of individual samples was measured with the Qubit dsDNA High Sensitivity kit (Thermo Fisher Scientific, #Q32854) kit to check if concentrations were roughly equal, and all samples were pooled at equal volume. The DNA concentration of the final pooled library was measured with the Qubit dsDNA High Sensitivity kit. 785pM library with 2% PhiX Sequencing Control was loaded onto an Illumina Nextseq2000 P1 flow cell and sequenced with 2x300bp paired-end read settings.

#### RNA quantification with droplet digital PCR

RNA expression data for *SMN2-FL* RNA from blood was available from Wadman et al*.*^8^ RNA expression data for *SMN2-FL* and *SMN2Δ7* from fibroblasts was available from Signoria et al.^57^: RNA was extracted from PAXgene blood RNA tubes (BD Biosciences) or fibroblasts with the RNeasy mini kit (Qiagen, 74104). Total RNA was treated with DNase I (Thermo Scientific, EN0521). 100ng RNA, as measured by spectrophotometer (Nanodrop 2000,Thermo Scientific), was used for cDNA synthesis using the High-capacity cDNA reverse transcription kit (Applied Biosystems, 4368814). Primers and probes used for quantification of *SMN2-FL*, *SMN2Δ7, SMN-AS1* and *TBP* (housekeeping gene for normalization) were obtained from IDT or Thermo Scientific (Table S5).^9^^,^^10^^,^^14^^,^^57^ For *SMN2-FL* and *SMN2Δ7*, reactions of 22μL contained: 1μL cDNA, 0.05μL *SMN*-specific probe (100μM), 0.05μL *TBP* probe (100μM), 1μL of forward and reverse *SMN*- and *TBP*-specific primers (10μM), 11μL of 2x ddPCR Supermix for probes (no dUTP, BioRad, 186-3024) and 5.9μL of RNase/DNase-free water. Droplets were generated using a QX200 Automated droplet generator (BioRad 1864101). PCR was run in a Bio-Rad T100 thermal cycler (95°C for 10 min, followed by 40 cycles of 95°C for 30 sec and 61.1°C for 1 min; followed by 98°C for 10 min; ramp rate 2°C/sec). Droplets were analyzed after amplification using a QX200 droplet reader (Bio-Rad 1864003). Expression levels of *SMN2-FL* and*SMN2Δ7* were normalized to *TBP* expression using QuantaSoft Software (Bio-Rad 1864011). Quantification of *SMN-AS1* was performed with the same protocol, with the following exceptions: 500ng of RNA was used for cDNA generation, using gene-specific (Table S5) and poly-dT primers. 5μL cDNA was used in the PCR reaction. All experiments were run in technical triplicates.

#### ONT data processing

ONT sequencing data was generated previously from 31 SMA patients^23^: in summary, three library preps with 1.3 μg HMW DNA each were made with the ligation sequencing kit (ONT, SQK-LSK109) per sequencing run. The library preps were sequenced on a FLO-MIN106 flow cell on a GridION (ONT) with MinKNOW v21.02.5-22.12.5 (ONT) and FAST basecalling for 72 hours, with a nuclease flush and reloading a new library prep every 24 hours. Adaptive sampling^90^^,^^91^ was used within MinKNOW, with a combined target FASTA file^23^ of the 30 Mb region surrounding the *SMN* locus (GRCh38 chr5:55,000,000-85,000,000) and six resolved alleles of the *SMN* locus downloaded from the study of Vollger et al.^92^^,^^93^ One of the 31 patients of this study was excluded due to a partial deletion of *SMN1* exon 1-6, another patient was excluded due to unavailability of clinical data, resulting in 29 patients for further analysis (Table S1). Raw sequencing data was basecalled with Guppy v6.1.2 with the SUP model^75^ (dna_r9.4.1_450bps_modbases_5mc_cg_sup.cfg) and mapped to the T2T-CHM13 reference genome masked for a ∼172kb region surrounding *SMN2* (chr5:70,772,138-70,944,284).^23^ If data from both blood and fibroblasts of the same patient was available, this data was not merged. Polyploid haplotype phasing was performed with HapSMA v1.0.0^23^^,^^76^. Methylation calling was performed for full bam files and per-haplotype bam files with modbam2bed v1.0 (ONT^77^) with options -e -m 5mC -r chr5:71375000-71425000 --cpg. Methylation calls from forward and reverse strands were merged per CpG site. Methylation percentage was calculated from modbam2bed output with the formula: Nmod/(Nmod + Ncan) ∗ 100.

#### Illumina data processing

Bisulfite sequencing data was processed with the methylseq v2.6.0 workflow from nf-core,^78^ including raw data QC with FastQC, adapter sequence and quality trimming (Phred<20) with Trim Galore!, read alignment to the masked T2T-CHM13 reference genome^23^ with Bismark and extraction of methylation calls with Bismark. Default options were used, except for: --unmapped, --skip_deduplication and --save_align_intermeds. For each sample, the bismark.cov.gz file from the methylation_coverage output directory was loaded into R4.4.0^79^ and the data for all samples were merged into one dataset. Methylation percentage was used directly from the cov.gz file. The data was filtered to only contain CpG sites that are located on the intended amplicons and had a read depth (methylated reads + unmethylated reads) of 100x or more. When the C or G of a CpG site was a known SNV position,^25^ it was removed, to prevent any SNVs to be mistakenly interpreted as methylation changes, since unconverted DNA was not sequenced. Additional data filtering was applied before statistical testing with linear models, to reduce dimensionality: sites with little variation between samples (methylation percentage standard deviation <5%) were removed, and highly correlated sites with Spearman’s R>0.9 were treated as one site by averaging (Figure S5).

#### Visualization and exploration of methylation data

Methylation percentages were visualized per sample or per haplotype using the pheatmap v1.0.12 package^80^ in R v4.4.0. Row clustering was performed with the ward.D2 method and Euclidian distance. PCA was performed with the function PCA from the FactoMineR v2.11 package^81^ followed by the function fviz_pca_ind from package factoextra v1.0.7.^82^

#### DNA motif analyses

The sequence surrounding the tested CpG sites (20 bp upstream and 20 bp downstream) was extracted with bedtools v2.30.0.^84^ DNA binding motifs for CTCF were downloaded from CTCF_HUMAN.H11MO.0.A on hocomoco11.autosome.org.^31^ FIMO (https://meme-suite.org/meme/doc/fimo.html) ^32^ was used to scan the *SMN1/2* gene sequence for CTCF binding motifs. Sequence logos were generated using https://weblogo.threeplusone.com/create.cgi.^85^

### Quantification and statistical analysis

#### Statistics

Required sample size was calculated in R4.4.0 using the pwr v1.3-0 package^83^ using the pwr.f2.test function. To detect a medium effect size (Cohen’s f^2^=0.15)^94^ in a linear model with a power of 80%, alpha of 0.01 and assuming 4 regression degrees of freedom (corresponding to three covariates), a sample size of 115 is required. Differential methylation analysis was performed using linear models in R4.4.0 with the following formula^95^: dependent_variable ∼ independent_variable + covariates; using methylation percentage as dependent variable. For each independent variable (such as age at sampling or SMA type), one formula was made per CpG site, thus one p-value was calculated for every CpG site and corrected for multiple testing with the false discovery rate (FDR) method. Covariates used were age at sampling in years (except when age was independent variable),^56^^,^^96^ sex,^97^ library size per *SMN* copy and GQN. Continuous independent variables such as age at sampling were included in the model as numeric values, whereas factors with two levels were converted to 0 and 1. Ordinal independent variables such as *SMN2* and *NAIP* copy number were used as numeric values. SMA type was converted to numeric in the following way before using it as independent variable: type 1 to 0, type 2 to 1, type 3 to 2 and type 4 to 3. For testing concordance in ONT data, the data was divided into two groups due to small sample size: less severe with three copies of *SMN2* and type 2b or less severe and with four copies of *SMN2* and type 3b or less severe; more severe with three copies of *SMN2* and type 2a or more severe and with four copies of *SMN2* and type 3a or more severe. Volcano plots were made by plotting the -log10 of the adjusted p-value against the estimate of the regression coefficient belonging to the independent variable for each CpG site. Results of all differential methylation analyses including effect sizes for all CpG sites are denoted in Tables S6, S7, and S8. All other statistical test results are indicated in the figure legends. ‘n’ represents number of patients unless mentioned otherwise. Summary statistics for methylation percentage per CpG site in ONT and bisulfite sequencing datasets are denoted in Tables S9 and S10.

## Supplemental information

Document S1. Figure S1–S12, Table S1–S5

Table S6. Results of all statistical tests performed for differential methylation analyses, related to Figure 1 A.) Results for Figure 1D. B.) Results for Figure 1E. C.) Results for Figure 1F. D.) Results for Figure 1H. E.) Results for Figure 1I. F.) Results for Figure S2B. G.) Results for Figure S2C. H.) Results for Figure S2E. I.) Results for Figure S2F. J.) Results for Figure S2I. K.) Results for Figure S2J

Table S7. Results of all statistical tests performed for differential methylation analyses, related to Figure 3 A.) Results for Figure 3C. B.) Results for Figure 3D. C.) Results for Figure S9C

Table S8. Results of all statistical tests performed for differential methylation analyses, related to Figure 4 A.) Results for Figure 4C. B.) Results for Figure 4D. C.) Results for Figure 4G. D.) Results for Figure S10A. E.) Results for Figure S10B. F.) Results for Figure S11A. G.) Results for Figure S11B. H.) Results for Figure S11C. I.) Results for Figure S11D. J.) Results for Figure S11E. K.) Results for Figure S11F

Table S9. Summary statistics for methylation percentage per CpG site in the ONT sequencing dataset, related to Figure 1 A.) Summary statistics for blood. B.) Summary statistics for fibroblasts

Table S10. Summary statistics for methylation percentage per CpG site in the bisulfite sequencing dataset, related to Figure 2
