# Supplementary material for: Comprehensive analysis across SMN2 excludes DNA methylation as an epigenetic biomarker for spinal muscular atrophy
Source: iScience. 2025 Apr 17;28(5):112461. doi: 10.1016/j.isci.2025.112461 (PMC12084074; doi:10.1016/j.isci.2025.112461)
Supplement: Document S1. Figure S1–S12, Table S1–S5 [file mmc1.pdf]

**iScience, Volume *28***

## Supplemental information

# Comprehensive analysis across *SMN2* excludes DNA methylation as an epigenetic biomarker for spinal muscular atrophy

**Maria M. Zwartkruis, Joris V. Kortooms, Demi Gommers, Martin G. Elferink, Ilaria Signoria, Joyce van der Sel, Paul J. Hop, Ramona A.J. Zwamborn, Robin Geene, Jared W. Green, Hanneke W.M. van Deutekom, Wouter van Rheenen, Jan H. Veldink, Fay-Lynn Asselman, Renske I. Wadman, W. Ludo van der Pol, Gijs W. van Haaften, and Ewout J.N. Groen**

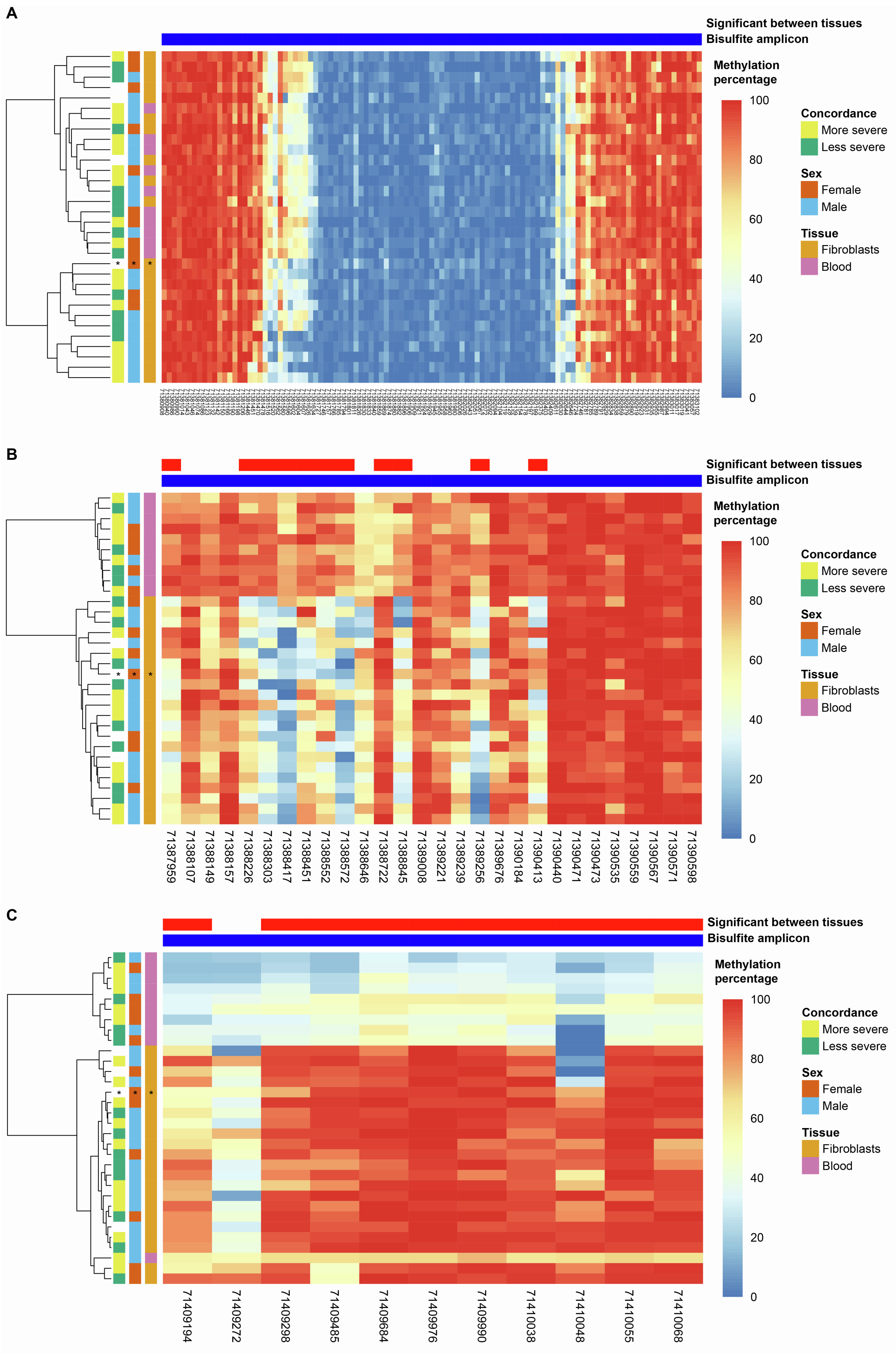
A
Significant between tissues
Bisulfite amplicon
Methylation percentage
100
80
60
40
20
0
Concordance
More severe
Less severe
Sex
Female
Male
Tissue
Fibroblasts
Blood
B
Significant between tissues
Bisulfite amplicon
Methylation percentage
100
80
60
40
20
0
Concordance
More severe
Less severe
Sex
Female
Male
Tissue
Fibroblasts
Blood
71387959
71388107
71388149
71388157
71388226
71388303
71388417
71388451
71388552
71388572
71388646
71388722
71388845
71389008
71389221
71389239
71389256
71389676
71390184
71390413
71390440
71390471
71390473
71390535
71390559
71390567
71390571
71390598
C
Significant between tissues
Bisulfite amplicon
Methylation percentage
100
80
60
40
20
0
Concordance
More severe
Less severe
Sex
Female
Male
Tissue
Fibroblasts
Blood
71409194
71409272
71409298
71409485
71409684
71409976
71409990
71410038
71410048
71410055
71410068

**Figure S1:** Heatmap of CpG DNA methylation in specific regions of the *SMN2* gene, Related to Fig. 1.
CpG DNA methylation in the promoter (A), *SMN-AS1* (B) and 3'UTR (C) regions in SMA patients with a homozygous *SMN1* deletion and one patient with one copy of *SMN1* with a pathogenic mutation (indicated with an asterisk (*)). Each row represents one patient, each column represents one CpG site (positions on T2T-CHM13 chromosome 5 are listed below the heatmap). Hierarchical clustering with the ward.D2 method was performed on the rows. Patient characteristics are shown left of the heatmap. Sites at which methylation significantly differs between tissues (Figure 1D, $p_{adj}$<0.01) and the bisulfite amplicons (Figure 2A) are shown above the heatmaps.

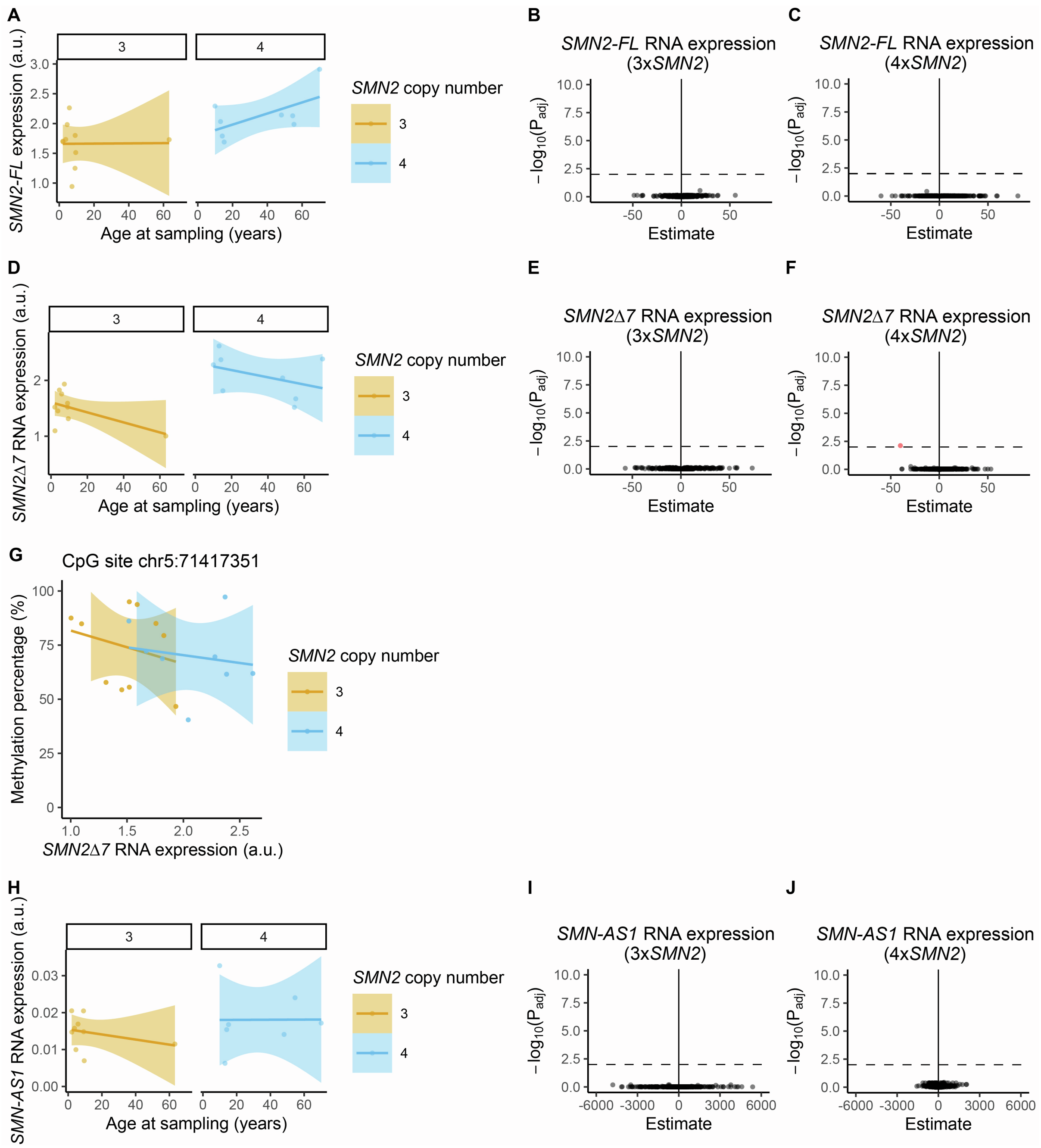


**Figure S2:** Differential methylation analysis for *SMN2-FL, SMN2Δ7 and SMN-AS1* RNA expression in fibroblasts, Related to Fig. 1.

**(A)** *SMN2-FL* RNA expression in fibroblasts with three or four *SMN2* copies. Lines represent linear models between age and *SMN2-FL* RNA expression, shading represents the 95% confidence interval of the linear models.

**(B-C)** Differential methylation analysis for different amounts of *SMN2-FL* RNA expression in SMA patients with three *SMN2* copies (B, n=10) or four *SMN2* copies (C, n=8) as shown in (A). No CpG sites were significantly associated with *SMN2-FL* RNA expression ($p_{adj}$<0.01).

**(D)** *SMN2Δ7* RNA expression in fibroblasts with three or four *SMN2* copies. Lines represent linear models between age and *SMN2Δ7* RNA expression, shading represents the 95% confidence interval of the linear models.

**(E-F)** Differential methylation analysis for different amounts of *SMN2Δ7* RNA expression in SMA patients with three *SMN2* copies (E, n=10) or four *SMN2* copies (F, n=8) as shown in (D). One CpG

site (T2T-CHM13 chr5:71,417,315) was significantly associated with *SMN2Δ7* RNA expression in the 4x*SMN2* group ($p_{adj}<0.01$).

**(G)** DNA methylation percentage at T2T-CHM13 chr5:71,417,315 plotted against *SMN2Δ7* RNA expression for patients with three or four *SMN2* copies. Lines represent linear models between *SMN2Δ7* RNA expression and methylation percentage, shading represents the 95% confidence interval of the linear models.

**(H)** *SMN-AS1* RNA expression in fibroblasts with three or four *SMN2* copies. Lines represent linear models between age and *SMN-AS1* RNA expression, shading represents the 95% confidence interval of the linear models.

**(I-J)** Differential methylation analysis for different amounts of *SMN-AS1* RNA expression in SMA patients with three *SMN2* copies (**I**, n=9) or four *SMN2* copies (J, n=7) as shown in (H). No CpG sites were significantly associated with *SMN-AS1* RNA expression ($p_{adj}<0.01$).

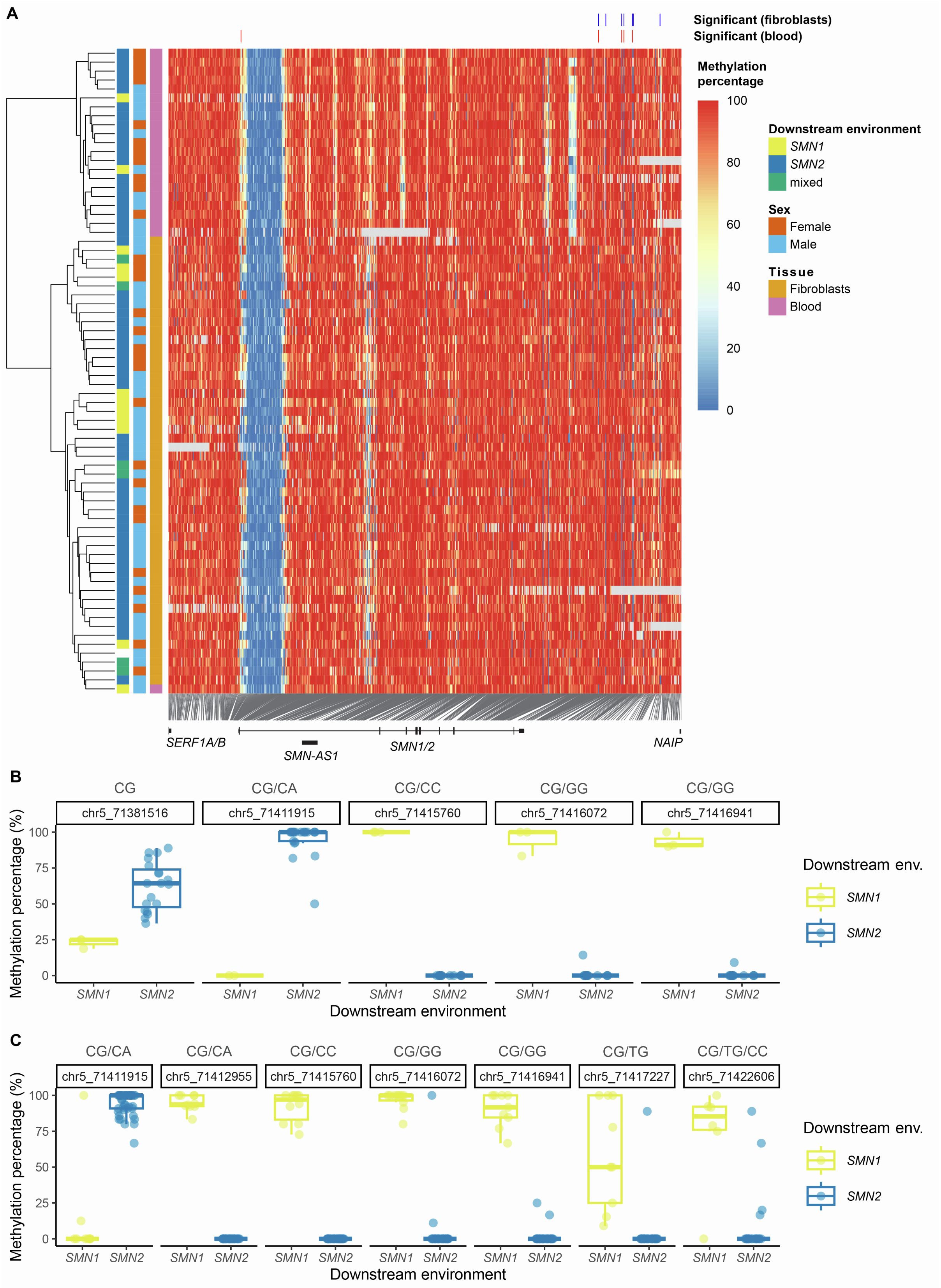


**Figure S3:** Per-haplotype analysis of DNA methylation in Oxford Nanopore Technologies (ONT) sequencing data of SMA patients, Related to Fig. 1.

**(A)** Heatmap of DNA methylation per haplotype, determined by ONT sequencing at T2T-CHM13 coordinates chr5:71,375,000-71,425,000, containing 710 CpG sites. 72 out of 108 haplotypes with less than 25% NA are included. Hierarchical clustering was performed according to the ward.D2

method. Sites that were differentially methylated between haplotypes with downstream *SMN1* versus *SMN2* environment (Fig. 1H-I) are shown at the top of the figure.

**(B-C)** Methylation percentage at CpG sites that were differentially methylated between different downstream environments in blood (B) and fibroblasts (C). Many of these sites are known SNV sites [S1]; the possible nucleotide changes are indicated above each site. Data are represented as median ± IQR (box). Whiskers represent 1.5 IQR.

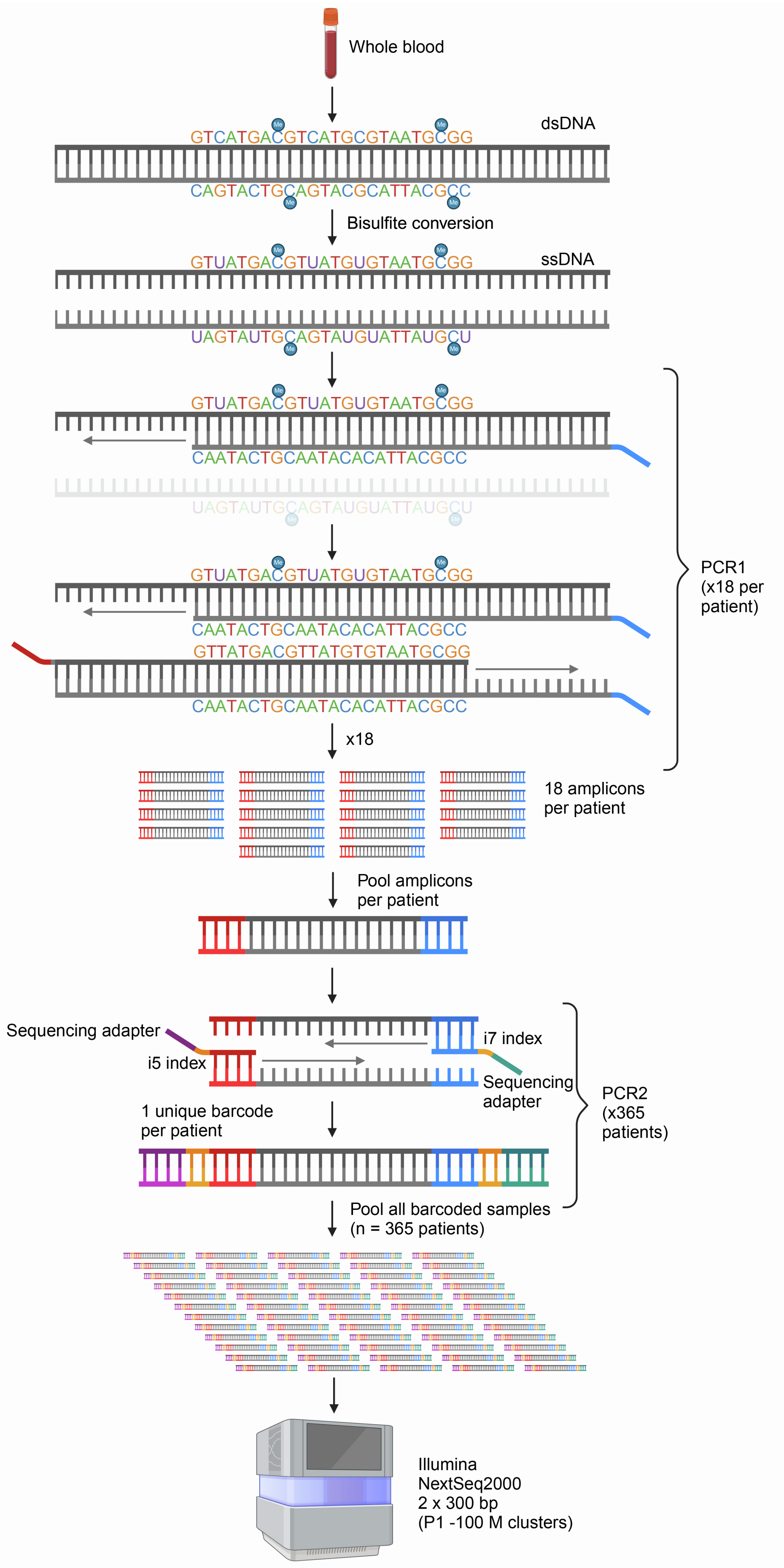

Whole blood
dsDNA
GTCATGACGTCATGCGTAATGCGG
CAGTACTGCAGTACGCATTACGCC
Bisulfite conversion
ssDNA
GTUATGACGTUATGUGTAATGCGG
UAGTAUTGCAGTAUGUATTAUGCU
GTUATGACGTUATGUGTAATGCGG
CAATACTGCAATACACATTACGCC
UAGTAUTGCAGTAUGUATTAUGCU
GTUATGACGTUATGUGTAATGCGG
CAATACTGCAATACACATTACGCC
GTTATGACGTTATGTGTAATGCGG
CAATACTGCAATACACATTACGCC
x18
PCR1 (x18 per patient)
18 amplicons per patient
Pool amplicons per patient
Sequencing adapter
i5 index
i7 index
Sequencing adapter
1 unique barcode per patient
PCR2 (x365 patients)
Pool all barcoded samples (n = 365 patients)
Illumina NextSeq2000 2 x 300 bp (P1 -100 M clusters)

**Figure S4**: Bisulfite sequencing methods, Related to Fig. 2.
Double-stranded DNA was isolated from whole blood and bisulfite-converted, resulting in single-stranded DNA. The top strand of the converted DNA was amplified by PCR, using primers with an overhang compatible with the sequencing adapters (Illumina DNA/RNA UD Indexes). 18 such PCRs were performed per patient and all amplicons were pooled. For each patient, sequencing adapters were ligated to the amplicons in a second round of PCR. Samples from 365 patients were pooled and sequenced on an Illumina NextSeq2000. Created with Biorender.com.

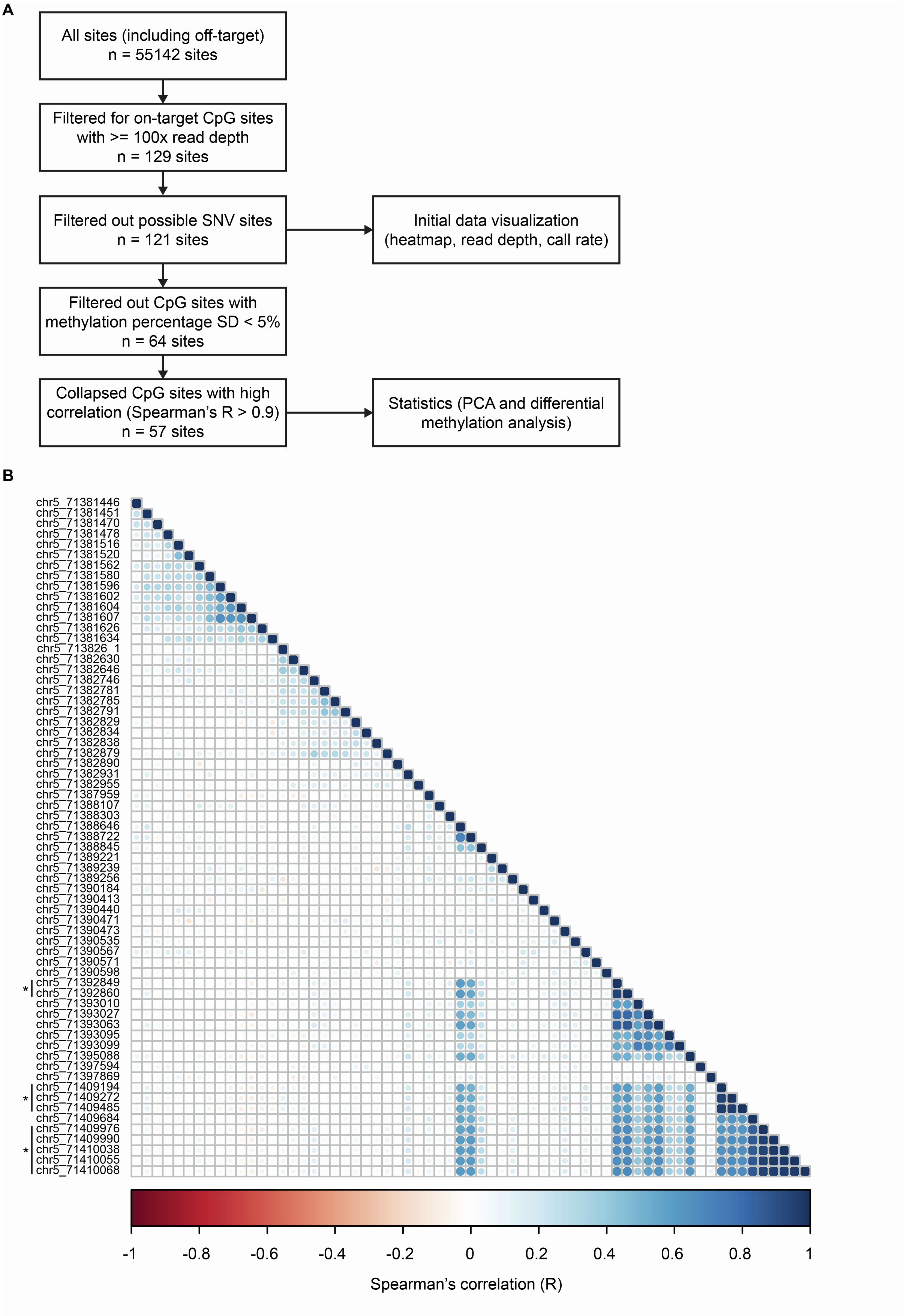


**Figure S5:** Dimensionality reduction by filtering of CpG sites, Related to Fig. 2.

**(A)** CpG sites were filtered for mapping on the intended targets, a minimum read depth of 100x, not being a known SNV site [S2] and not having a low standard deviation (SD). Lastly, sites with high Spearman correlation (R>0.9) as shown in (B), were condensed into one site by taking their mean.
**(B)** Correlation plot showing the Spearman correlation between methylation at the tested CpG sites, used for the last filtering step of (A). Sites with Spearman R>0.9 are indicated by an asterisk (*).

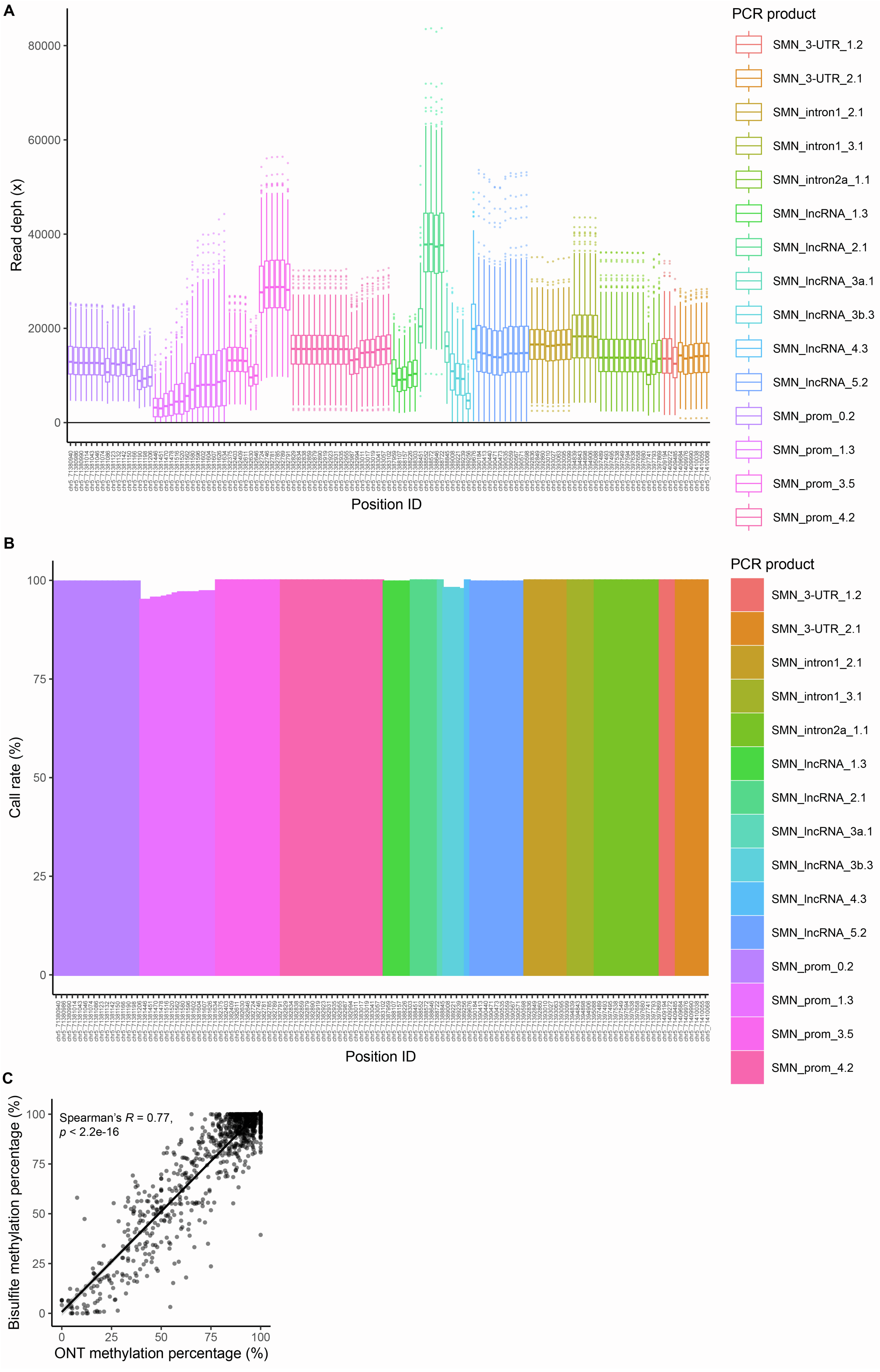
A
Read depth (x)
80000
60000
40000
20000
0
Position ID
PCR product
SMN_3-UTR_1.2
SMN_3-UTR_2.1
SMN_intron1_2.1
SMN_intron1_3.1
SMN_intron2a_1.1
SMN_lncRNA_1.3
SMN_lncRNA_2.1
SMN_lncRNA_3a.1
SMN_lncRNA_3b.3
SMN_lncRNA_4.3
SMN_lncRNA_5.2
SMN_prom_0.2
SMN_prom_1.3
SMN_prom_3.5
SMN_prom_4.2
B
Call rate (%)
100
75
50
25
0
Position ID
PCR product
SMN_3-UTR_1.2
SMN_3-UTR_2.1
SMN_intron1_2.1
SMN_intron1_3.1
SMN_intron2a_1.1
SMN_lncRNA_1.3
SMN_lncRNA_2.1
SMN_lncRNA_3a.1
SMN_lncRNA_3b.3
SMN_lncRNA_4.3
SMN_lncRNA_5.2
SMN_prom_0.2
SMN_prom_1.3
SMN_prom_3.5
SMN_prom_4.2
C
Spearman's R = 0.77,
p < 2.2e-16
Bisulfite methylation percentage (%)
100
75
50
25
0
ONT methylation percentage (%)

**Figure S6:** Performance of bisulfite sequencing, Related to Fig. 2.
**(A)** Median read depth per CpG site represented by the midline of boxplots, after filtering for sites with at least 100x read depth and filtering out possible SNV sites. The box represents the interquartile range. Data are represented as median ± IQR (box). Whiskers represent 1.5 IQR.
**(B)** Call rate per CpG site: percentage of samples that have ≥100x read depth, giving a valid methylation call.
**(C)** Spearman correlation between ONT and bisulfite methylation percentages, determined from nine samples for which both data types were available; $p<2.2e-16$, Spearman's $R=0.77$. The black line represents the linear model between ONT methylation percentage and bisulfite methylation percentage, grey shading represents the 95% confidence interval of the linear model.

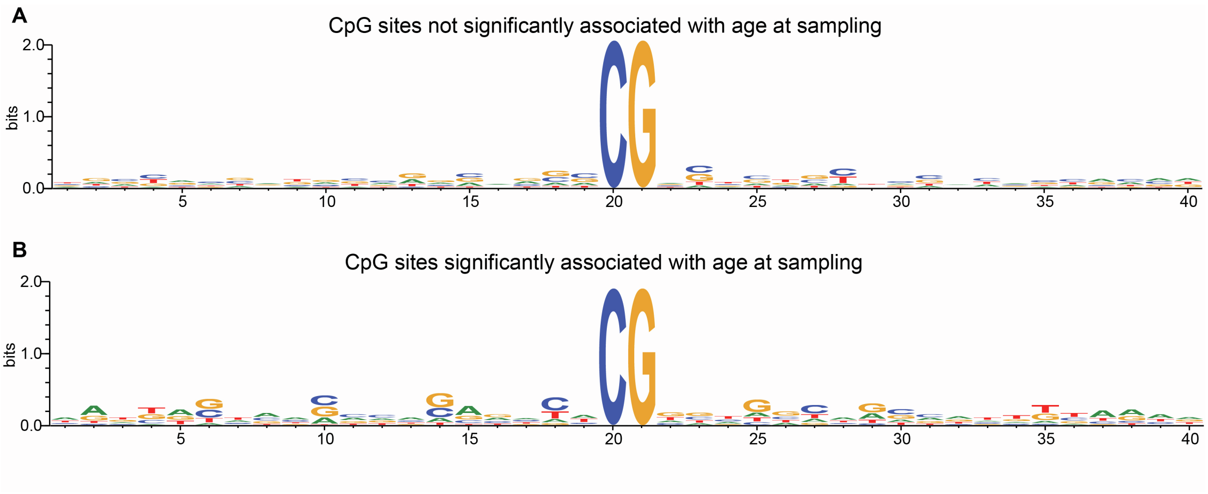


**Figure S7:** Nucleotide motif surrounding CpG sites that are not significantly associated with age (n=42 sites, A) and CpG sites that are significantly associated with age (n=22 sites, B), Related to Fig. 3.

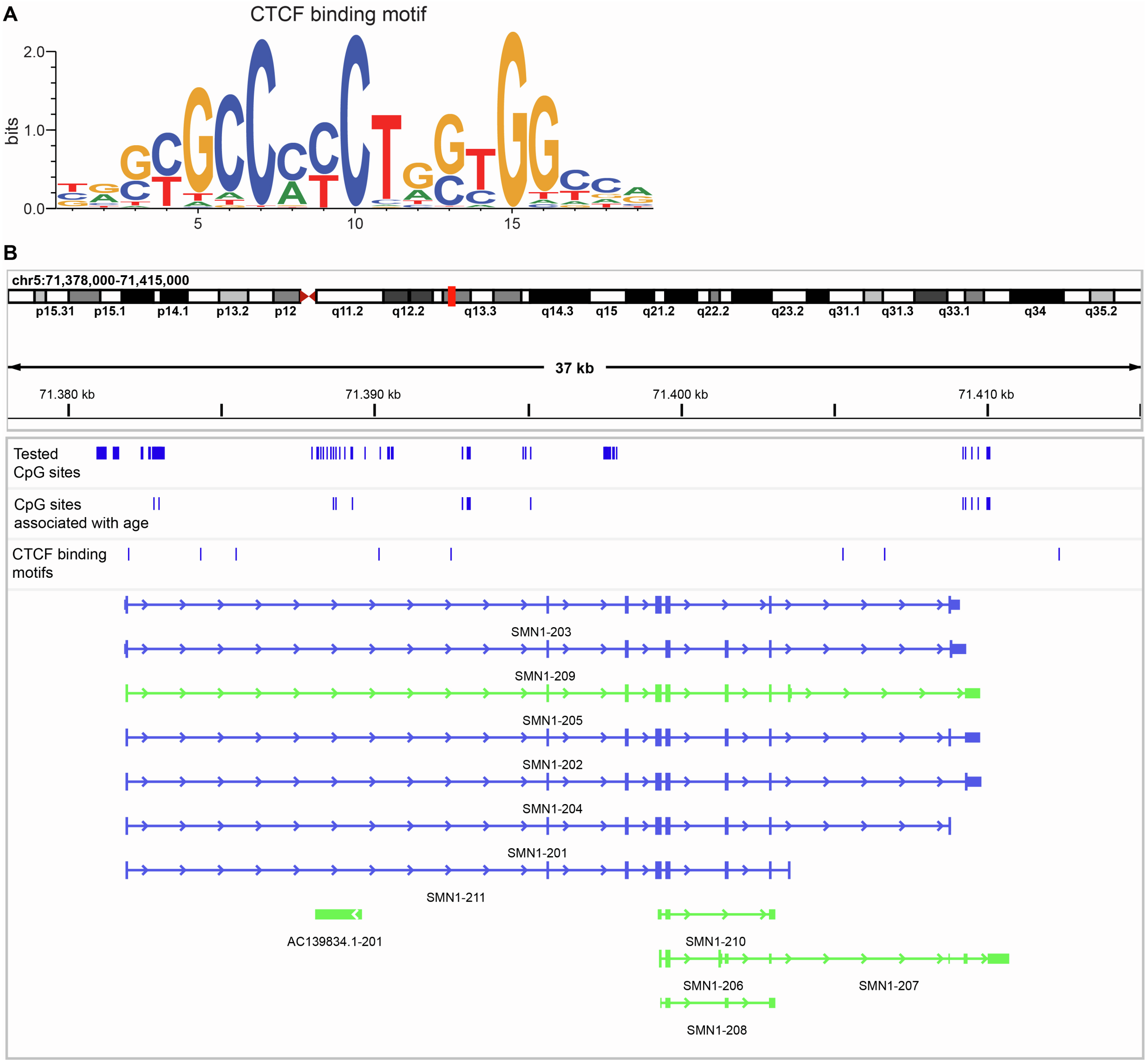


**Figure S8:** CTCF binding motifs on the *SMN* gene, Related to Fig. 3.
**(A)** CTCF binding motif.
**(B)** IGV snapshot of the *SMN1* gene on T2T-CHM13 reference genome, *SMN1-202* being the canonical transcript. CTCF binding motifs do not overlap with the CpG sites associated with age (Figure 3E).

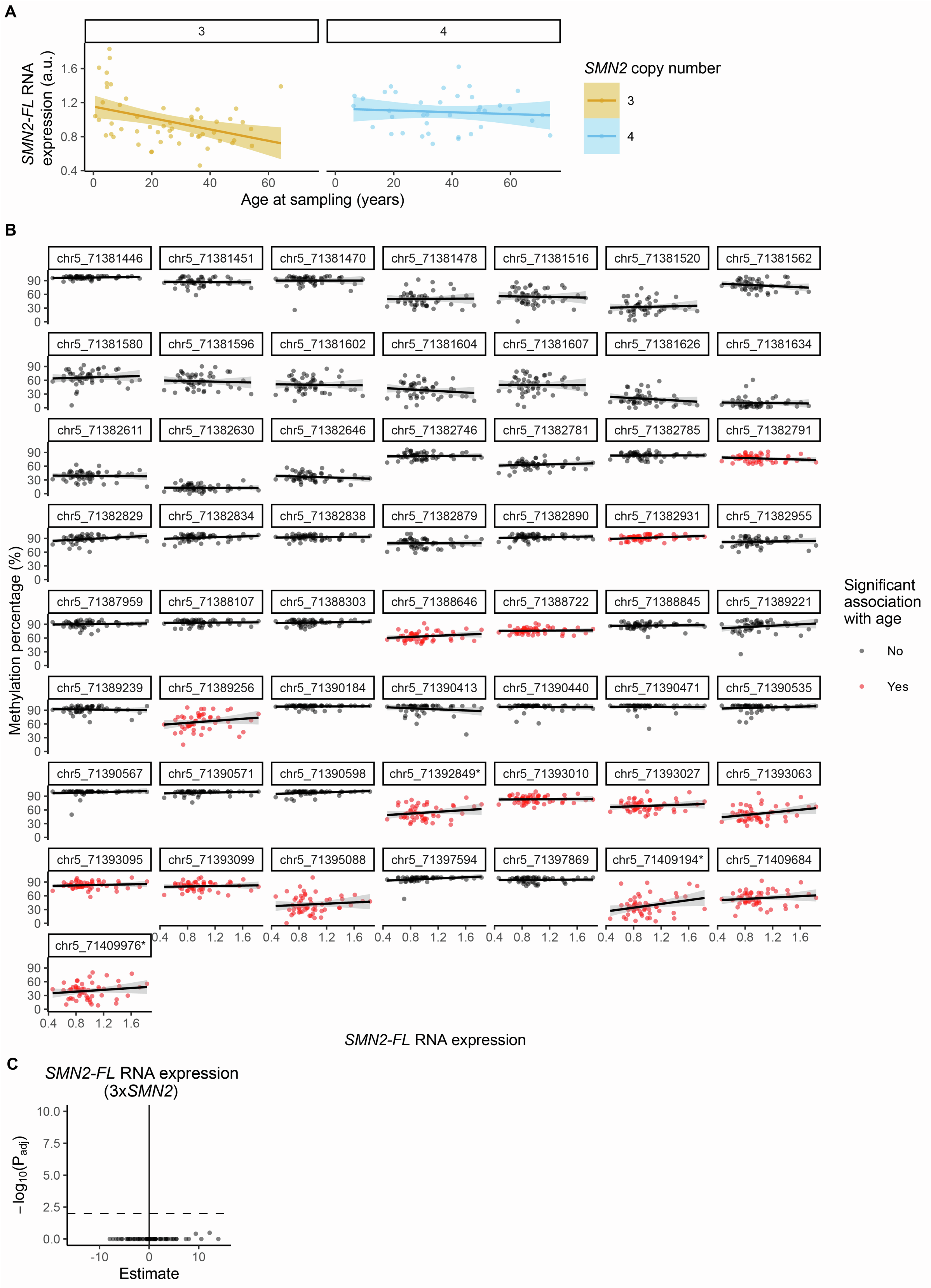


**Figure S9:** Association between age, *SMN2-FL* RNA expression and DNA methylation in *SMN2*, Related to Fig. 3.

**(A)** *SMN2-FL* RNA expression [S3] was associated with age at sampling in patients with three copies of *SMN2* (simple linear regression, used model: SMN2-FL=1.153-0.006714*(age), adjusted R-squared: 0.1407, F(1,51)=9.514, p=0.003289) but not in patients with four copies of *SMN2* (simple

linear regression, used model: SMN2-FL=1.129-0.001071*(age), adjusted R-squared: -0.01986, F(1,36)=0.2795, p=0.6003). Lines represent linear models between age and *SMN2-FL* RNA expression, shading represents the 95% confidence interval of the linear models.

**(B)** DNA methylation percentage plotted against *SMN2-FL* RNA expression for patients with three *SMN2* copies. CpG sites at which DNA methylation is associated with age are shown in red. Collapsed sites are indicated with an asterisk (*). Black lines represent linear models between *SMN2-FL* RNA expression and methylation percentage, grey shading represents the 95% confidence interval of the linear models.

**(C)** Differential methylation analysis for different amounts of *SMN2-FL* RNA expression in SMA patients with three *SMN2* copies (n=53) as shown in (B). No CpG sites were significantly associated with *SMN2-FL* RNA expression ($p_{adj}$<0.01).

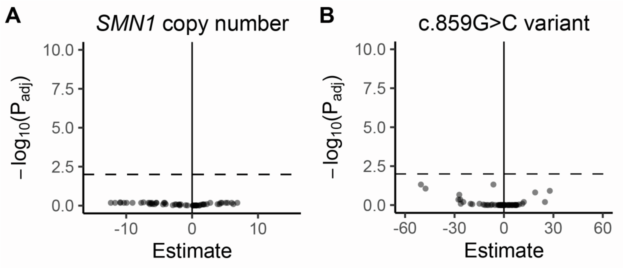


**Figure S10:** Differential methylation analysis for patients with known functional genetic variants, Related to Fig. 4.

**(A)** Differential methylation analysis for patients with *SMN1* with a pathogenic mutation (n=4) versus patients with a homozygous *SMN1* deletion with similar *SMN2* copy number of two or three (n=230). No CpG sites were significantly associated with presence of the *SMN1* gene ($p_{adj}$<0.01).

**(B)** Differential methylation analysis for patients with the c.859G>C variant in *SMN2* (n=2) versus patients without this variant and the same *SMN2* copy number of two (n=15). No CpG sites were significantly associated with presence of the c.859G>C variant ($p_{adj}$<0.01).

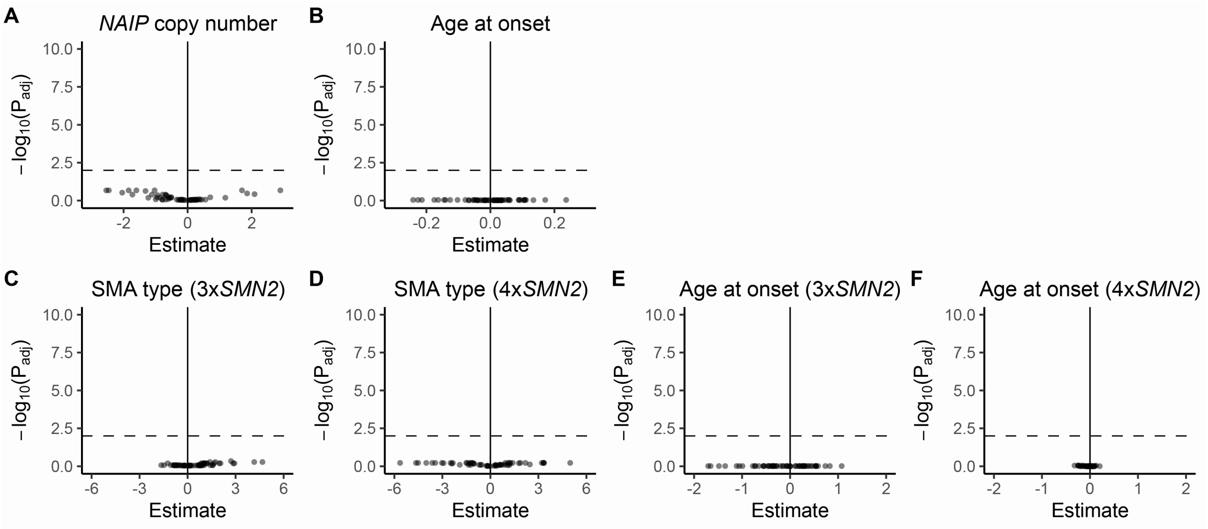


**Figure S11:** Differential methylation analysis, Related to Fig. 4.

**(A)** Differential methylation analysis between patients with different *NAIP* copy numbers, ranging from zero to four (n=359). No differentially methylated sites were found ($p_{adj}$<0.01).

**(B)** Differential methylation analysis between patients with different ages at onset, ranging from 0 to 43 years old (n=333). No differentially methylated sites were found ($p_{adj}$<0.01).

**(C-D)** Differential methylation analysis between different SMA types in patients with three *SMN2* copies (C, n=215) and four *SMN2* copies (D, n=122). No differentially methylated sites were found ($p_{adj}$<0.01).

**(E-F)** Differential methylation analysis between different ages at onset in patients with three *SMN2* copies (E, n=204) and four *SMN2* copies (F, n=109). No differentially methylated sites were found ($p_{adj}$<0.01).

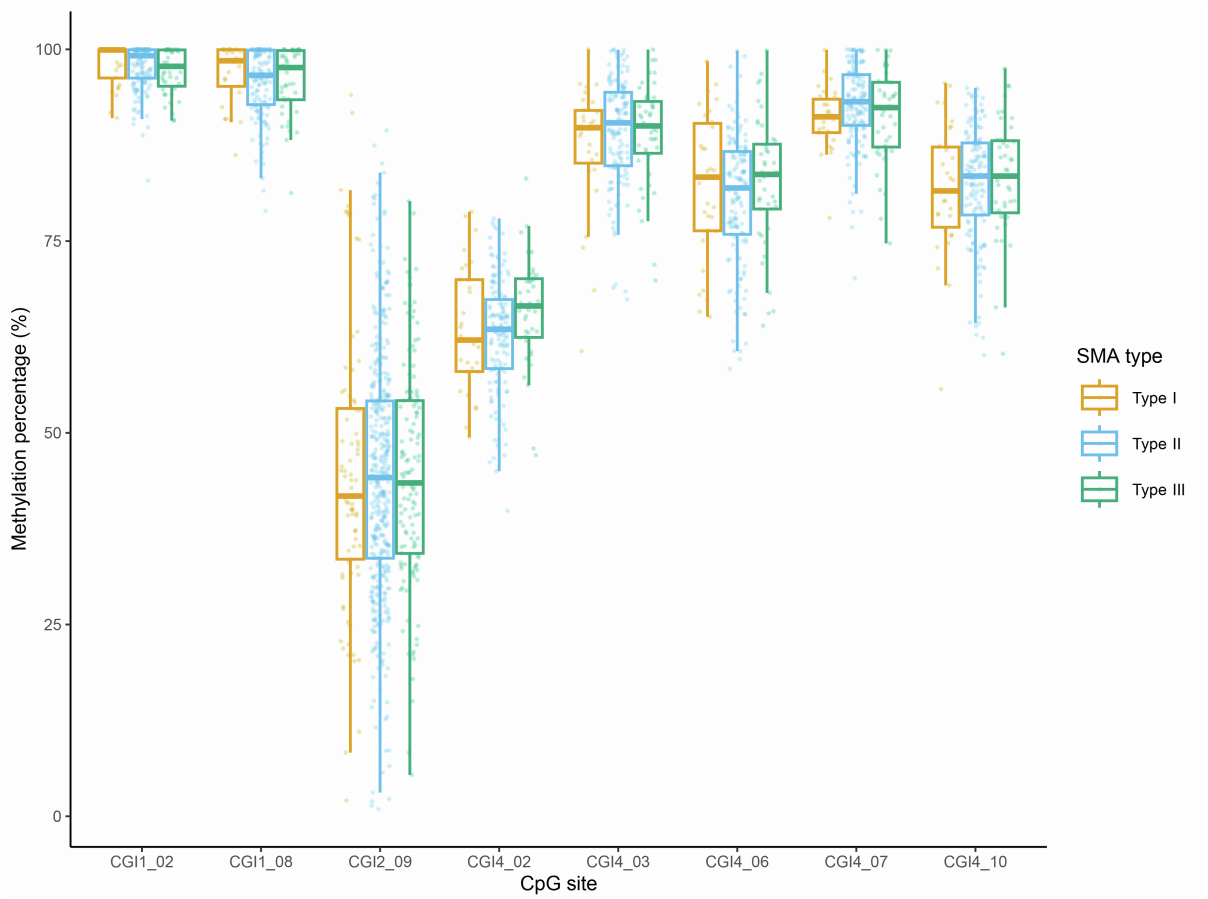


**Figure S12:** DNA methylation at sites reported to be associated with SMA disease severity in the study by Cao *et al.* [S4], in patients with three copies of *SMN2* and SMA type 1 (n=30), type 2 (n=138) and type 3 (n=43), Related to Fig. 4.

For each CpG site, the Kruskal-Wallis rank sum test was performed with FDR multiple testing correction: CGI1_02 (chr5:71,381,014): $p_{adj}$=1; CGI1_08 (chr5:71,381,150): $p_{adj}$=1; CGI2_09 (chr5:71,381,602, chr5:71,381,604 and chr5:71,381,607): $p_{adj}$=1; CGI4_02 (chr5:71,382,781): $p_{adj}$=0.2804; CGI4_03 (chr5:71,382,829): $p_{adj}$=1; CGI4_06 (chr5:71,382,879): $p_{adj}$=1; CGI4_07 (chr5:71,382,890): $p_{adj}$=0.60656; CGI4_10 (chr5:71,382,955): $p_{adj}$=1. Data are represented as median ± IQR (box). Whiskers represent 1.5 IQR.

**Table S1:** Baseline characteristics of SMA patients sequenced with ONT sequencing, Related to Fig. 1.

| | Blood | | Fibroblasts | | | |
|---|---|---|---|---|---|---|
| | 3x*SMN2* | 4x*SMN2* | 2x*SMN2* | 3x*SMN2* | 4x*SMN2* | 5x*SMN2* |
| **Total** | 6 | 4 | 3 | 10 | 8 | 1 |
| **Sex** | | | | | | |
| Male | 3 (50.0%) | 2 (50.0%) | 1 (33.3%) | 6 (60.0%) | 7 (87.5%) | 1 (100%) |
| Female | 3 (50.0%) | 2 (50.0%) | 2* (66.7%) | 4 (40.0%) | 1 (12.5%) | 0 (0%) |
| **SMA type** | | | | | | |
| Type 1b | 0 (0%) | 0 (0%) | 2 (66.7%) | 0 (0%) | 0 (0%) | 0 (0%) |
| Type 1c | 2 (33.3%) | 0 (0%) | 0 (0%) | 1 (10.0%) | 0 (0%) | 0 (0%) |
| Type 2a | 2 (33.3%) | 0 (0%) | 0 (0%) | 6 (60.0%) | 1 (12.5%) | 0 (0%) |
| Type 2b | 1 (16.7%) | 2 (50.0%) | 0 (0%) | 1 (10.0%) | 1 (12.5%) | 0 (0%) |
| Type 3a | 1 (16.7%) | 0 (0%) | 1* (33.3%) | 1 (10.0%) | 1 (12.5%) | 0 (0%) |
| Type 3b | 0 (0%) | 1 (25.0%) | 0 (0%) | 1 (10.0%) | 3 (37.5%) | 1 (100%) |
| Type 4 | 0 (0%) | 1 (25.0%) | 0 (0%) | 0 (0%) | 2 (25.0%) | 0 (0%) |
| **Age at onset (years)** | | | | | | |
| Mean (SD) | 0.708 (0.244) | 7.00 (9.11) | 0.403 (0.528) | 1.77 (2.75) | 11.0 (9.03) | 15.5 (NA) |
| Median [Min, Max] | 0.646 [0.417, 1.00] | 3.00 [1.50, 20.5] | 0.208 [0, 1.00] | 0.813 [0.500, 9.50] | 12.7 [0.833, 24.5] | 15.5 [15.5, 15.5] |
| **Age at sampling (years)** | | | | | | |
| Mean (SD) | 12.3 (14.4) | 45.1 (13.6) | 11.4 (17.7) | 11.7 (18.3) | 35.0 (24.3) | 21.1 (NA) |
| Median [Min, Max] | 6.71 [4.50, 41.5] | 45.3 [30.0, 59.7] | 1.92 [0.417, 31.8] | 6.58 [2.08, 63.3] | 31.6 [9.92, 70.1] | 21.1 [21.1, 21.1] |

*Includes one patient with one *SMN1* gene copy with a pathogenic mutation.

**Table S2:** Baseline characteristics of SMA patients sequenced with bisulfite sequencing, with *SMN1* with a known pathogenic mutation, Related to Fig. 2.

| | 2x*SMN2* (n=2) | 3x*SMN2* (n=2) | Overall (n=4) |
|---|---|---|---|
| **Sex** | | | |
| Male | 1 (50.0%) | 1 (50.0%) | 2 (50.0%) |
| Female | 1 (50.0%) | 1 (50.0%) | 2 (50.0%) |
| **SMA type** | | | |
| Type 1 | 0 (0%) | 1 (50.0%) | 1 (25.0%) |
| Type 3 | 2 (100%) | 1 (50.0%) | 3 (75.0%) |
| **Age at onset (years)** | | | |
| Mean (SD) | 2.50 (2.12) | 1.00 (0.707) | 1.75 (1.55) |
| Median [Min, Max] | 2.50 [1.00, 4.00] | 1.00 [0.500, 1.50] | 1.25 [0.500, 4.00] |
| **Age at sampling (years)** | | | |
| Mean (SD) | 40.4 (14.1) | 30.8 (42.9) | 35.6 (26.7) |
| Median [Min, Max] | 40.4 [30.4, 50.4] | 30.8 [0.474, 61.2] | 40.4 [0.474, 61.2] |

**Table S3:** Baseline characteristics of SMA patients sequenced with bisulfite sequencing, with a known c.859G>C variant in *SMN2*, Related to Fig. 2.

| | **2x*SMN2* (n=2)** | **Overall (n=2)** |
|---|---|---|
| **Sex** | | |
| Male | 1 (50.0%) | 1 (50.0%) |
| Female | 1 (50.0%) | 1 (50.0%) |
| **SMA type** | | |
| Type 2 | 1 (50.0%) | 1 (50.0%) |
| Type 3 | 1 (50.0%) | 1 (50.0%) |
| **Age at onset (years)** | | |
| Mean (SD) | 4.83 (5.89) | 4.83 (5.89) |
| Median [Min, Max] | 4.83 [0.667, 9.00] | 4.83 [0.667, 9.00] |
| **Age at sampling (years)** | | |
| Mean (SD) | 36.7 (17.9) | 36.7 (17.9) |
| Median [Min, Max] | 36.7 [24.1, 49.3] | 36.7 [24.1, 49.3] |

**Table S4:** Overview of bisulfite amplicons, related to Fig. 2.

| Amplicon name | Forward primer sequence | Reverse primer sequence | Annealing temperature (ºC) | DNA input per 10µL PCR reaction (ng) | Amplicon coordinates on T2T-CHM13 *SMN1* | Amplicon coordinates on T2T-CHM13 *SMN2* | Number of CpG sites |
|---|---|---|---|---|---|---|---|
| SMN_prom_0.2 | TCGTCGGCAGCGTCAGATGTGTATAAGAGACAGTATTTTAGTTTGGGTGATAGAGTAATAT | GTCTCGTGGGCTCGGAGATGTGTATAAGAGACAGAAACTCCTACTAAAAATTTAAAACCTAC | 61.4 | 5 | chr5:71380875-71381350 | chr5:70838191-70838666 | 17 |
| SMN_prom_1.3 | TCGTCGGCAGCGTCAGATGTGTATAAGAGACAGTAAAATAAATAATAAATAAGGTTGGGGG | GTCTCGTGGGCTCGGAGATGTGTATAAGAGACAGTCATTTCACTATATTATATTTCCTTTCC | 58.1 | 30 | chr5:71381211-71381698 | chr5:70837851-70838330 | 14 |
| SMN_prom_2.2 | TCGTCGGCAGCGTCAGATGTGTATAAGAGACAGATAATATAGTGAAATGAAAGGATTGAGA | GTCTCGTGGGCTCGGAGATGTGTATAAGAGACAGAAAAAAAAATAAACAAAATAAACTACTAC | 55.6 | 5 | chr5:71381682-71382233 | chr5:70837316-70837867 | 44 |
| SMN_prom_3.5 | TCGTCGGCAGCGTCAGATGTGTATAAGAGACAGTGTTATTAATAGAGTGAAAGTATTTGGA | GTCTCGTGGGCTCGGAGATGTGTATAAGAGACAGCCCAAAATACTAAAATTACAAACTTAAA | 58.1 | 5 | chr5:71382327-71382824 | chr5:70836725-70837222 | 13 |
| SMN_prom_4.2 | TCGTCGGCAGCGTCAGATGTGTATAAGAGACAGTTTAGGTTTTGGAAGGTTTAGGG | GTCTCGTGGGCTCGGAGATGTGTATAAGAGACAGTTTAAATAAAATTCCTTAAAATATACCT | 55.6 | 5 | chr5:71382673-71383133 | chr5:70836416-70836876 | 19 |
| SMN_intron1_1.1 | TCGTCGGCAGCGTCAGATGTGTATAAGAGACAGAGGGGGAAGTGTTATATATTTTAAAATA | GTCTCGTGGGCTCGGAGATGTGTATAAGAGACAGAACTAAAATAAAAAAATCCCTTAAACTT | 53.6 | 10 | chr5:71385508-71386053 | chr5:70833497-70834042 | 9 |
| SMN_lncRNA_1.3 | TCGTCGGCAGCGTCAGATGTGTATAAGAGACAGAATAGATATAGTGGTTTTTTGTGATTTT | GTCTCGTGGGCTCGGAGATGTGTATAAGAGACAGTCAACAACAAAAAAATTCAATTAAAAAAT | 55.6 | 5 | chr5:71387871-71388345 | chr5:70831205-70831679 | 6 |
| SMN_lncRNA_2.1 | TCGTCGGCAGCGTCAGATGTGTATAAGAGACAGAGTTTTTTATGTTTGTTGGGTATAATAT | GTCTCGTGGGCTCGGAGATGTGTATAAGAGACAGAACTTCCAACTTCCTTATCTAAATATAT | 58.1 | 5 | chr5:71388354-71388807 | chr5:70830743-70831196 | 6 |
| SMN_lncRNA_3a.1 | TCGTCGGCAGCGTCAGATGTGTATAAGAGACAGTTTTGAGAGAGGAGGTAAAAAAAGATTA | GTCTCGTGGGCTCGGAGATGTGTATAAGAGACAGATTATACAAACAAATCCTTAACCTAAAC | 55.6 | 5 | chr5:71388517-71388975 | chr5:70830575-70831033 | 1 |
| SMN_lncRNA_3b.3 | TCGTCGGCAGCGTCAGATGTGTATAAGAGACAGTAAGGATTTGTTTGTATAATAAAAGGTT | GTCTCGTGGGCTCGGAGATGTGTATAAGAGACAGAATCCTTATACAAAAACAAACCAAATTT | 61.4 | 10 | chr5:71388956-71389455 | chr5:70830095-70830594 | 4 |
| SMN_lncRNA_4.3 | TCGTCGGCAGCGTCAGATGTGTATAAGAGACAGGGTTTGTTTTTGTATAAGGATTTTTAAT | GTCTCGTGGGCTCGGAGATGTGTATAAGAGACAGTATCATACAAATAAAACTCCCAAATAAC | 55.6 | 5 | chr5:71389434-71389903 | chr5:70829647-70830116 | 1 |

| | | | | | | | |
|---|---|---|---|---|---|---|---|
| SMN_lncRNA_5.2 | TCGTCGGCAGCGTCAGATGTGTATAAGAGACAGGATATGTTATTAGGATTTTTTGTGGTT | GTCTCGTGGGCTCGGAGATGTGTATAAGAGACAGTAAAATCAAAAATTCAAAACCAACCTAA | 62.9 | 10 | chr5:71390145-71390644 | chr5:70828906-70829405 | 10 |
| SMN_intron1_2.1 | TCGTCGGCAGCGTCAGATGTGTATAAGAGACAGAATTTTATTTTATAGTATAAATTGGATTT | GTCTCGTGGGCTCGGAGATGTGTATAAGAGACAGTAAAAAAACCCCTTCTCTACTAAAAATA | 55.6 | 5 | chr5:71392682-71393138 | chr5:70826412-70826868 | 8 |
| SMN_intron1_3.1 | TCGTCGGCAGCGTCAGATGTGTATAAGAGACAGTATGTTTGTAATTTTTATATTTGGGAGG | GTCTCGTGGGCTCGGAGATGTGTATAAGAGACAGAAAATACTATTCTAACCTCAAAACCTAT | 58.1 | 1.25 | chr5:71394810-71395294 | chr5:70824256-70824740 | 6 |
| SMN_intron2a_1.1 | TCGTCGGCAGCGTCAGATGTGTATAAGAGACAGATTAATAGTTTTAGAAGTGTGTTTGTAG | GTCTCGTGGGCTCGGAGATGTGTATAAGAGACAGTCTTCCTAATATTCATAACCCTTTAAAT | 61.4 | 5 | chr5:71397422-71397921 | chr5:70821629-70822128 | 13 |
| SMN_intron6_2.1 | TCGTCGGCAGCGTCAGATGTGTATAAGAGACAGTTATTGGATATATTGAAAGAAGAAAAAT | GTCTCGTGGGCTCGGAGATGTGTATAAGAGACAGAAAAAACAAAACAAAACACAAAACTAA | 55.6 | 5 | chr5:71406895-71407431 | chr5:70812119-70812655 | 16 |
| SMN_3-UTR_1.2 | TCGTCGGCAGCGTCAGATGTGTATAAGAGACAGATGGGATAATTTAGGTATATTGTATTGT | GTCTCGTGGGCTCGGAGATGTGTATAAGAGACAGTTATCCAATATCATTCAAAATCTAATCC | 61.4 | 1.25 | chr5:71409095-71409544 | chr5:70810002-70810451 | 4 |
| SMN_3-UTR_2.1 | TCGTCGGCAGCGTCAGATGTGTATAAGAGACAGTAAATGGTTTAATAAAATGTATGTGAGG | GTCTCGTGGGCTCGGAGATGTGTATAAGAGACAGACAAAACTTAATAAAACATAATACCACA | 58.1 | 5 | chr5:71409656-71410119 | chr5:70809428-70809891 | 7 |

**Table S5:** Overview of primers and probes used for ddPCR, Related to Fig. 1.

| **Target** | **Primer/probe** | **Sequence** |
|---|---|---|
| *SMN2-FL* | Forward primer | AC ATG AGT GGC TAT CAT ACT GGC TA |
| | Probe | 5'-6FAM ATA TGG GTT TTA GAC AAA A MGB-3' |
| | Reverse primer | AAT GTG AGC ACC TTC CTT CTT TTT |
| *SMN2Δ7* | Forward primer | GG CTA TCA TAC TGG CTA TTA TAT GGA A |
| | Probe | 5'-6FAM CTG GCA TAG AGC AGC ACT AAA TGA CAC CAC MGB-3' |
| | Reverse primer | TCC AGA TCT GTC TGA TCG TTT CTT |
| *SMN-AS1* | Forward primer | CCT TTC TCT CCG TGC AGC |
| | Probe | 6-FAM-ATG TTG TGA GTT GTT GGG GTT G-MGB |
| | Reverse primer | CGA CTG GAG CAC GAG GAC ACT GA |
| | Reverse transcription primer | CGA CTG GAG CAC GAG GAC ACT GAA GAT AAG GAA GCT GGA AGC |
| *TBP* | Forward primer | CGT GGT TCG TGG CTC TCT |
| | Probe | 5'-HEX ATC CCA AGC -ZEN- GGT TTG CTG-3' |
| | Reverse primer | GCC CGA AAC GCC GAA TAT |

## SUPPLEMENTAL REFERENCES


[S1] Zwartkruis, M.M., Elferink, M.G., Gommers, D., Signoria, I., Blasco-Pérez, L., Costa-Roger, M., Sel, J. van der, Renkens, I.J., Green, J.W., Kortooms, J.V., et al. (2024). Long-read sequencing identifies copy-specific markers of *SMN* gene conversion in spinal muscular atrophy. Preprint at medRxiv. https://doi.org/10.1101/2024.07.16.24310417.

[S2] Chen, X., Harting, J., Farrow, E., Thiffault, I., Kasperaviciute, D., Hoischen, A., Gilissen, C., Pastinen, T., and Eberle, M.A. (2023). Comprehensive *SMN1* and *SMN2* profiling for spinal muscular atrophy analysis using long-read PacBio HiFi sequencing. Am. J. Hum. Genet. *110*, 240–250. https://doi.org/10.1016/j.ajhg.2023.01.001.

[S3] Wadman, R.I., Jansen, M.D., Stam, M., Wijngaarde, C.A., Curial, C.A.D., Medic, J., Sodaar, P., Schouten, J., Vijzelaar, R., Lemmink, H.H., et al. (2020). Intragenic and structural variation in the *SMN* locus and clinical variability in spinal muscular atrophy. Brain Commun. *2*, fcaa075. https://doi.org/10.1093/braincomms/fcaa075.

[S4] Cao, Y., Qu, Y., He, S., Li, Y., Bai, J., Jin, Y., Wang, H., and Song, F. (2016). Association between *SMN2* methylation and disease severity in Chinese children with spinal muscular atrophy. J. Zhejiang Univ.-Sci. B *17*, 76–82. https://doi.org/10.1631/jzus.B1500072.
